# MBOAT1 Promotes Glioma Progression Through Enhancing Ferroptosis Resistance and Immunosuppressive Microenvironment

**DOI:** 10.1002/cns.70785

**Published:** 2026-02-16

**Authors:** Junqi Fan, Qingqing Huang, Lanxin Bao, Xueran Chen, Zhiyou Fang, Haifeng Shu

**Affiliations:** ^1^ Department of Neurosurgery The General Hospital of Western Theater Command Chengdu China; ^2^ Department of Anesthesiology The General Hospital of Western Theater Command Chengdu China; ^3^ Hefei Cancer Hospital of CAS; Institute of Health and Medical Technology, Hefei Institutes of Physical Science Chinese Academy of Sciences (CAS) Hefei Anhui China

**Keywords:** ferroptosis, glioma, immune infiltration, MBOAT1, prognosis, single cell

## Abstract

**Background:**

Emerging evidence indicates that ferroptosis characterized by lipid peroxidation is becoming a promising therapeutic strategy in glioma. However, the role of the MBOAT family, key regulators of membrane phospholipids remodeling in ferroptosis, remains unexplored in glioma.

**Methods:**

We systematically analyzed the expression and clinical significance of MBOAT1 in glioma using TCGA, CGGA, GEO, and GTEx databases. Functional mechanisms were investigated through enrichment, single‐cell RNA sequencing, and immune infiltration analyses. We experimentally validated the oncogenic role of MBOAT1 in GBM through both in vivo and in vitro experiments.

**Results:**

MBOAT1 expression was elevated in glioma and correlated with increased grades and poor patient prognosis. Cox regression analysis identified MBOAT1 as an independent prognostic factor. Functional enrichment analysis and single cell RNA‐seq analysis revealed that MBOAT1 is associated with enhanced ferroptosis resistance. Furthermore, the immune infiltration analysis and cell communication analysis suggested that MBOAT1 promotes an immunosuppressive microenvironment. Experiments confirmed that overexpression of MBOAT1 promoted GBM cell proliferation, migration, invasion, and ferroptosis resistance, while its knockdown had the opposite effect.

**Conclusion:**

Our findings suggest that MBOAT1 promotes glioma progression by mediating ferroptosis resistance and is related to an immunosuppressive microenvironment, highlighting its potential as an independent prognostic biomarker and a promising therapeutic target.

## Graphical abstract

Integrating bulk and single‐cell RNA sequencing with functional experiments, we identify MBOAT1 as a key oncogenic driver in glioma. Our findings demonstrate that it promotes tumor progression by conferring resistance to ferroptosis and fostering an immunosuppressive microenvironment, thereby nominating it as a promising therapeutic target.

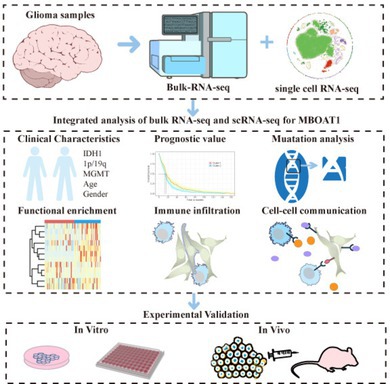

## 1 Introduction

Glioma is the most common primary malignant brain tumor in adults, accounting for approximately 23% of all primary brain and other central nervous system (CNS) tumors. Notably, up to 80% of gliomas are malignant [1]. According to the World Health Organization (WHO) classification, gliomas are graded from I to IV based on their malignancy: grades I and II are classified as low‐grade gliomas, whereas grades III and IV are considered high‐grade [2]. Glioblastoma multiforme (GBM), a grade IV glioma subtype, is characterized by aggressive growth and diffuse infiltration into surrounding brain tissue, which substantially limits treatment efficacy and negatively impacts patient outcomes [3]. Despite advances in GBM therapy, patients’ prognosis remains poor, largely due to treatment resistance [4]. Therefore, there is an urgent need for research aimed at developing novel therapeutic strategies for glioma.

Ferroptosis, a form of programmed cell death driven by accumulation of membrane lipid peroxidation and lethal reactive oxygen species (ROS), is increasingly implicated in glioma pathogenesis [5]. Evidence indicates that ferroptosis is not only the most enriched form of programmed cell death in glioma but also strongly associated with malignant progression, poor survival, and an immunosuppressive tumor microenvironment [6]. Furthermore, inducers of ferroptosis, including Erastin and RSL3, have been shown to directly initiate ferroptosis [7, 8] and enhance the susceptibility of glioma cells to radiotherapy or temozolomide (TMZ) treatment [9, 10, 11, 12]. Therefore, targeting ferroptosis represents a promising therapeutic strategy for glioma.

The peroxidation of polyunsaturated fatty acids (PUFAs) within membrane phospholipids is a central event of ferroptosis [13]. This process is facilitated by the esterification and incorporation of PUFAs into phospholipids, reactions catalyzed by acyl‐CoA synthetase long‐chain family member 4 (ACSL4) and lysophosphatidylcholine acyltransferase 3 (LPCAT3, also known as MBOAT5). Conversely, monounsaturated fatty acids (MUFAs) are less oxidizable than PUFAs. Therefore, cellular mechanisms that enhance the uptake, synthesis, and incorporation of MUFAs into phospholipids in place of PUFAs universally suppress lipid peroxidation and confer resistance to ferroptosis [14, 15, 16, 17].

Membrane‐bound O‐acyltransferase (MBOAT) family proteins represent a superfamily of integral transmembrane enzymes [18]. MBOAT family members mediate re‐acylation to remodel membrane properties and store fatty acids for bioactive lipid synthesis, including MBOATs1, 2, 5, and 7 [19]. Nonetheless, research on this family of proteins in cancer remains very limited. New evidence suggests sex hormone signaling inhibits ferroptosis through MBOAT1/2‐mediated phospholipid remodeling in ER^+^ breast cancer and AR^+^ prostate cancer, respectively [20]. However, the molecular mechanisms by which MBOATs mediate membrane phospholipid remodeling to promote glioma progression remain unexplored. Thus, this study aims to investigate the role of MBOAT1 in glioma progression, focusing on its regulation of ferroptosis resistance and the immunosuppressive tumor microenvironment (TME). Our findings identify MBOAT1 as a novel prognosis predictor and highlight its potential as a therapeutic target in glioma.

## 2 Methods

### 2.1 Data Acquisition

The bulk‐RNA‐seq and corresponding clinical data 942 glioma cases were sourced from The Cancer Genome Atlas (TCGA; https://portal.gdc.cancer.gov) database. Additionally, the two independent glioma cohorts from the Chinese Glioma Genome Atlas (*n* = 325 and *n* = 693; CGGA; http://www.cgga.org.cn) database were utilized. The normal tissue bulk‐RNA‐seq data were obtained from the Genotype‐Tissue Expression (GTEx; https://www.gtexportal.org) database. The single cell RNA‐seq data of 18 glioma patients (GSE182109) [21] were obtained from Gene Expression Omnibus (GEO, http://www.ncbi.nlm.nih.gov/geo/) database. The bulk‐RNA‐seq data of glioma cell lines were acquired from the (CELL; https://sites.broadinstitute.org/ccle) database.

### 2.2 Survival Prognosis Analysis

Kaplan–Meier analyses were conducted using data from the TCGA cohorts, CGGA_325 and CGGA_693 to evaluate the association between MBOAT1 expression and overall survival (OS) in glioma patients. The Receiver Operating Characteristic (ROC) curves were plotted using the “timeROC” R package to assess the prognostic accuracy of MBOAT1 expression. Also, nomogram construction and Cox regression analysis were conducted using the “Survival” and “rms” R packages in both the CGGA and TCGA glioma cohorts.

### 2.3 Mutation and Copy Number Variation Analysis

Based on the median expression of MBOAT1, patients in the TCGA cohort were divided into two groups. The “maftools” R package was then utilized to visualize the mutation status and copy number variations across these groups with different clinical characteristics.

### 2.4 Immune Infiltration Analysis

To investigate the relationship between MBOAT1 expression and immune cell infiltration in glioma, the “ESTIMATE” R package was used to assess the stromal, immune, and ESTIMATE scores for groups with MBOAT1‐high and MBOAT1‐low groups. The proportional infiltration of 22 immune cell types was assessed using “CIBERSORT” *R* package. To further explore the immune infiltration status in glioma, we used “IOBR” *R* package, which integrated 8 published algorithms for quantitative TME: CIBERSORT, TIMER, xCell, MCPcounter, ESITMATE, EPIC, IPS, quanTIseq [22].

### 2.5 Single Cell RNA‐Seq Analysis

The single‐cell RNA‐seq datasets were preprocessed by filtering out mitochondrial and ribosomal genes. Following the removal of low quality and doublet cells, we performed normalization, clustering, and batch‐effect correction using the “Seurat” and “Harmony” *R* packages, respectively. Then the cluster‐specific marker genes were identified and visualized. Subsequently, cell–cell communication between distinct cell subpopulations, particularly focusing on macrophages and microglia, was explored using the “CellChat” R package.

### 2.6 Function Enrichment Analysis

The TCGA cohort was divided into two groups based on the median expression of MBOAT1. Following differential gene expression analysis, the “ClusterProfiler” R package was employed to perform Gene Ontology (GO) and Kyoto Encyclopedia of Genes and Genomes (KEGG) enrichment analyses. Gene Set Enrichment Analysis (GSEA) and single sample Gene Set Enrichment Analysis (ssGSEA) were also conducted to explore and identify relevant signaling pathways.

### 2.7 Cell Culture

The glioma cell lines U87MG and LN18 were obtained from Cellcook (Guangzhou, China). Cells were cultured in Dulbecco's Modified Eagle Medium (Gibco, USA) supplemented with 10% fetal bovine serum (Gibco, USA) and 1% penicillin–streptomycin (Procell, China), under an atmosphere of 5% CO_2_ at 37°C.

### 2.8 Plasmid and Lentiviral or siRNA Transduction

Lentiviral plasmids for MBOAT1 overexpression and nonspecific control sequence were purchased from Genechem (Shanghai, China). Similarly, plasmid constructs for transient MBOAT1 overexpression and nonspecific control sequence were also purchased from Genechem (Shanghai, China). MBOAT1 specific and nonspecific control siRNAs were synthesized by GenePharma (Shanghai, China).

### 2.9 Wound Healing Assay

U87MG and LN18 cells were transfected and seeded into six‐well plates. Upon reaching approximately 90% confluence, a wound was created in each well using a 20 μL pipette tip. After washing with PBS to remove dislodged cells, fresh medium was added. The wound closure was imaged under a microscope at 0 h, 12 h and 24 h, and the images taken at 24 h were used for statistical analysis to calculate the average migration distance.

### 2.10 Transwell Invasion Assay

Cell invasive capacity was evaluated using 24‐well transwell chambers pre‐coated with Matrigel (LABSELECT, China), following the manufacturer's protocol. Transfected U87MG and LN18 cells were cultured in the upper chamber in serum‐free medium and allowed to invade for 24 h. Cells that invaded to the lower surface were fixed and stained with crystal violet.

### 2.11 Cell Proliferation Assay

Proliferation was assessed using an EdU assay kit (RIBOBIO, China). Transfected U87MG and LN18 cells were seeded in six‐well plates and grown to approximately 80% confluence before EdU labeling, performed according to the manufacturer's instructions.

### 2.12 Reactive Oxygen Species (ROS) Measurement

Cellular ROS levels were measured using the Cellular Reactive Oxygen Species Detection Assay Kit (Abcam, UK). Transfected U87MG and LN18 cells were seeded in six‐well plates and cultured to reach approximately 80% confluence, followed by treatment with RSL3 for 24 h. ROS fluorescence was measured at 648 nm using a confocal microscope.

### 2.13 Cell Viability Assay

Cell viability was assessed using a CCK‐8 kit (MedChemExpress, China). U87MG and LN18 cells were plated in 96‐well plates at a density of 5000 cells per well. After 24 h of transfection with plasmids and siRNA, cells were then exposed to RSL3 for another 24 h. Absorbance was measured according to the manufacturer's instructions to determine cell viability.

### 2.14 Colony Formation Assay

Cells were seeded in 6‐well plates at 2000 cells per well and transfected with plasmids or siRNA for 24 h, followed by RSL3 treatment. After 2 weeks culture, cells were fixed in 4% PFA at room temperature for 30 min, washed three times with PBS, and stained with 0.1% crystal violet (Solario, China). Colonies were visually counted and imaged.

### 2.15 Real‐Time PCR

Total RNA was extracted utilizing TRIzol (Invitrogen, USA). cDNA was synthesized from the extracted RNA using the PrimeScript RT Master Mix (TAKARA, Japan). Quantitative real‐time PCR was performed on a CFX96 Real‐time PCR Detection System, with primer sequences for ACTB (F: 5′‑CATGTACGTTGCTATCCAGGC‑3′; R: 5′‑CTCCTTAATGTCACGCACGAT‑3′) and MBOAT1 (F: 5′‑GTTTCGCATCTACTTACGTCCTG‑3′; R: 5′‑GCACATTAACACCAGCACAAAA‑3′).

### 2.16 Western Blot

Total protein was extracted using RIPA buffer (Solarbio, China) containing phenylmethylsulfonyl fluoride (Solarbio, China) and protease inhibitor cocktail (MedChemExpress, China). Protein concentration was determined using a BCA Protein Assay Kit (Solarbio, China). Equal amounts of proteins were separated by SDS‐PAGE gels and transferred to PVDF membranes. Membranes incubated with primary antibodies against MBOAT1 (Cat No. 25615–1‐AP, Proteintech, 1:1000), GAPDH (Cat No. 60004–1‐Ig, Proteintech, 1:1000), and GPX4 (Cat No. ab125066, Abcam, 1:1000) were incubated.

### 2.17 Mice Xenograft Model

This study utilized female BALB/c nude mice for the in vivo experiments. This approach was selected for the initial functional validation of MBOAT1's core oncogenic mechanisms in glioma. Our rationale is supported by bioinformatic evidence indicating that MBOAT1 expression in human glioma samples shows no significant association with patient sex, nor any notable correlation with key sex hormone receptors (ESR1, AR). These data suggest that the primary oncogenic role of MBOAT1 is independent of sex‐related factors in this context. The use of a single sex for this initial mechanistic investigation is consistent with the established methodology employed in our prior works on glioma models [23, 24] and aligns with practices in the field [25, 26, 27, 28, 29, 30]. We acknowledge that a comprehensive understanding of MBOAT1 biology will require future investigations in both male and female models.

Four‐week‐old female BALB/c nude mice were obtained from SPF Biotechnology Co. Ltd. (Beijing, China). MBOAT1‐overexpression U87MG cell line was established using lentivirus transduction purchased from Genechem (Shanghai, China) and selected with puromycin. Subcutaneous transplantation (1 × 10^7^cells per mouse) was performed under the guidance. When tumor volume reached 50 ~ 100 mm3, intraperitoneally injected with PBS or RSL3 (3 mg/kg) four times a week. Tumor volume was measured every 2 days. Tumor volume (cm^3^) = 0.5 × length × width^2^.

### 2.18 Statistical Analysis

GraphPad Prism version 9.0, ImageJ version 1.8.0.112 and R version 4.3.3 were used for statistics and visualization. The normality of data distribution was assessed using the Shapiro–Wilk test. Based on the normality assessment, appropriate statistical tests were selected as follows: for comparisons between two groups, an unpaired two‐tailed Student's *t*‐test was used for normally distributed data, and the Mann–Whitney U test was applied for non‐normally distributed data; for multiple group comparisons, one‐way analysis of variance (ANOVA) was employed for normally distributed data, while the Kruskal–Wallis test served as the nonparametric alternative. Categorical variables were analyzed using Fisher's exact test. For survival analysis, Kaplan–Meier curves were plotted and compared with the log‐rank test, while Cox proportional hazards regression model was used for multivariate survival analysis. Correlation between continuous variables was assessed via Pearson correlation analysis. Statistical significance was assigned as ***p < 0.001; **p < 0.01; *p < 0.05; ns (not significant) *p* > 0.05.

## 3 Results

### 3.1 Elevated MBOAT1 Expression Correlates With Patient Clinicopathologic Features in Glioma

**FIGURE 1:**
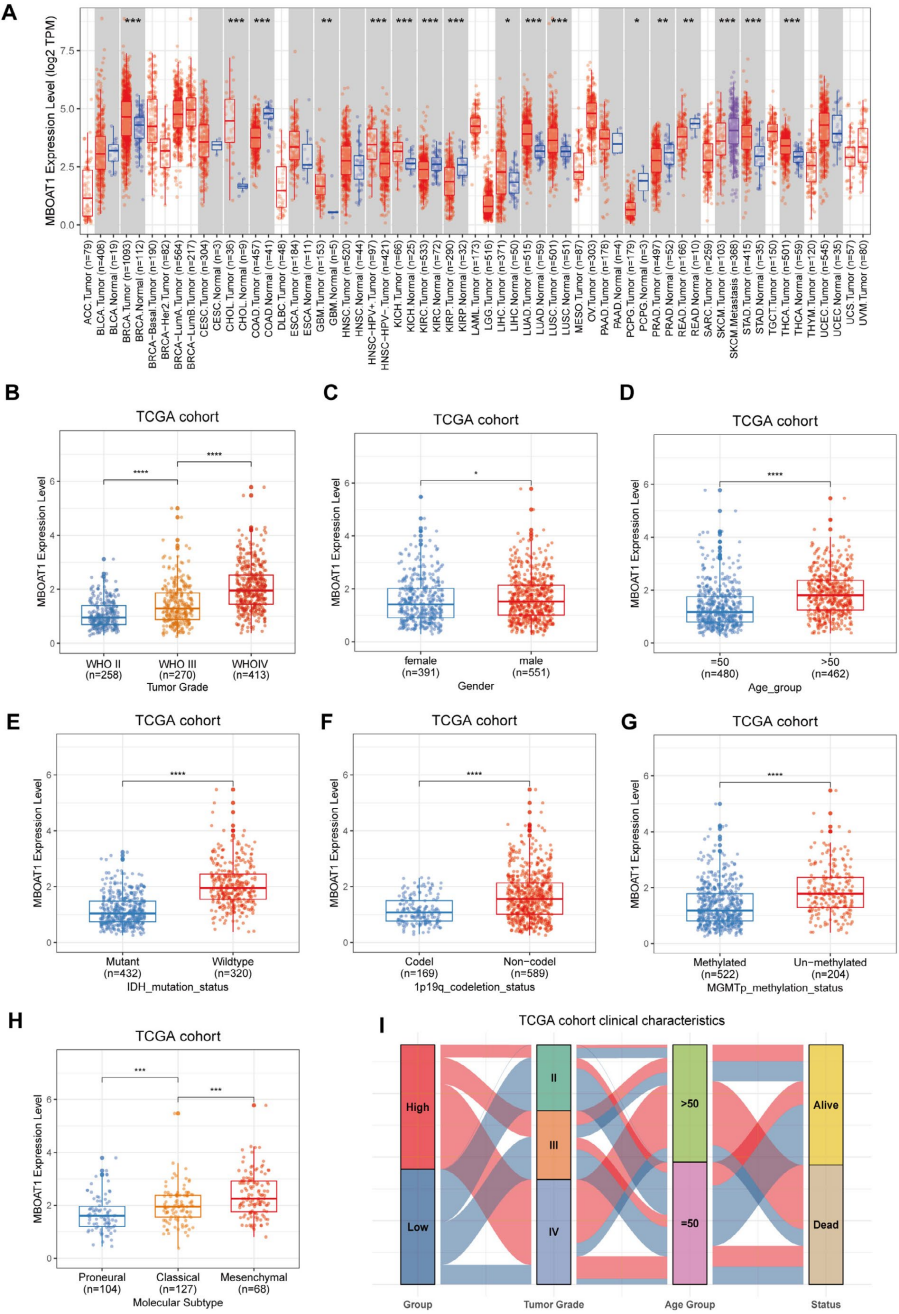
Correlation of MBOAT1 with clinical variables. (A) Expression of MBOAT1 in pan‐cancer from TCGA database. Box plots showing the association between MBOAT1 and various clinical variables in TCGA cohort, including WHO grade (B), gender (C), age (D), IDH mutation status (E), 1p19q codeletion status (F), MGMTp methylation status (G), Molecular Subtype (H). (I) The Sankey diagram demonstrates the relationships between clinical outcomes and MBOAT1 expression. Data are presented as mean ± SD. *p < 0.05; **p < 0.01; ***p < 0.001; ****p < 0.0001.

We initiated our study by analyzing MBOAT1, MBOAT2, MBOAT5, and MBOAT7 mRNA expression in pan‐cancer based on TIMER2.0 [31] database (http://timer.cistrome.org/). Among these members, MBOAT2 and MBOAT7 expression did not show significant upregulation in glioma (Figure S1A, B). In contrast, both MBOAT1 and MBOAT5 (LPCAT3) were markedly upregulated in glioma tissues (Figure S1C; Figure 1A). Since MBOAT5 is known to promote ferroptosis by facilitating the esterification of PUFAs into PE, we focused on MBOAT1 for further study. Subsequently, analysis of TCGA and GTEx databases also revealed higher levels of MBOAT1 expression in glioma compared to non‐tumor brain tissues (Figure S1D). Additionally, no significant association was observed between gender and the expression of MBOAT1 or MBOAT2 (Figure S2A).

To investigate the relationship between MBOAT1 expression and glioma malignancy. we first analyzed its distribution across WHO grades in the TCGA cohort. The results revealed stepwise increase in MBOAT1 expression with higher‐grade glioma (Figure 1B). Consistent results were reached when analyzing another two glioma cohorts from the CGGA database (CGGA_325, CGGA_693), showing increased MBOAT1 expression in higher‐grade gliomas (Figure S2B, C). Analysis of TCGA data revealed distinct correlation patterns: while MBOAT1 expression exhibited a weak association with patient gender (Figure 1C), elevated MBOAT1 expression showed significant correlations with multiple established poor prognostic indicators—including age > 50 years, IDH wild‐type status, 1p/19q non‐codeletion, MGMT promoter unmethylation, and mesenchymal GBM subtype (Figure 1D–H). The correlation between elevated MBOAT1 expression and adverse clinicopathological features was further validated in external CGGA cohorts (CGGA_325, CGGA_693), which also robustly associated it with a significantly increased risk of recurrence. (Figure S2D–I; Figure S3A–F). Within the morphological classification framework of gliomas, MBOAT1 expression levels are significantly elevated in Anaplastic Astrocytoma (AA), recurrent Anaplastic Astrocytoma (rAA), Glioblastoma (GBM) and recurrent Glioblastoma (rGBM) relative to other subtypes (Figure S3G–I). Collectively, these findings indicate MBOAT1 is upregulated in glioma, especially in GBM, and is associated with poor clinical outcome in glioma (Figure 1I; Figure S3J, K).

### 3.2 MBOAT1 Is an Independent Predictor of Patient Outcomes in Glioma

**FIGURE 2:**
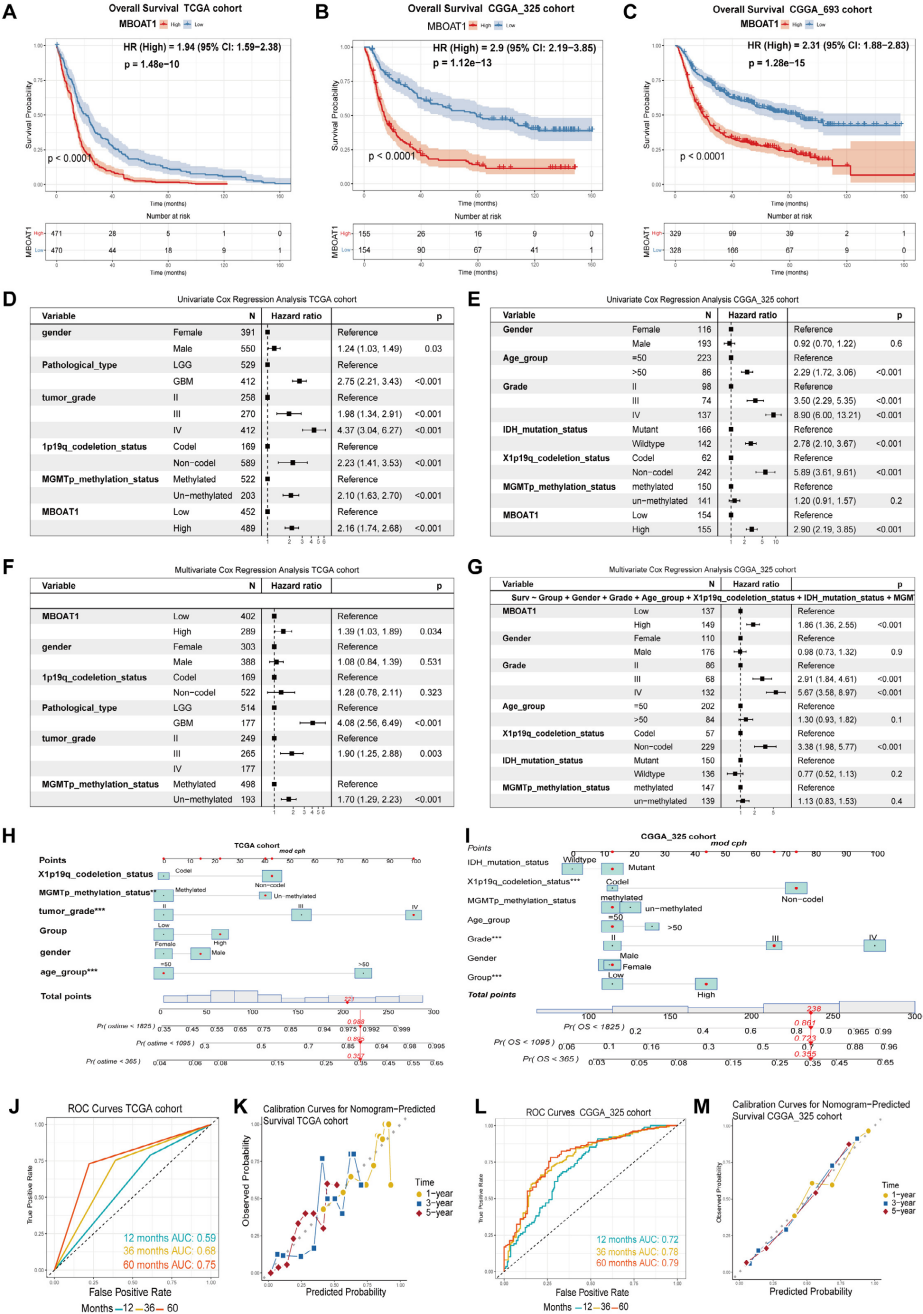
The prognostic value of MBOAT1 expression in TCGA and CGGA cohorts. (A–C) Kaplan–Meier analyses of OS in glioma patients from the TCGA, CGGA_325, and CGGA_693 databases. (D, E) Univariate Cox regression analysis of clinical pathological characteristics in glioma patients. (F, G) Multivariate Cox regression analysis of clinical pathological characteristics in glioma patients. A nomogram was utilized to predict the OS of glioma patients from the TCGA (H) and CGGA_325_cohort (I). (J, K) ROC curves to evaluate the predictive ability of MBOAT1 on survival time of patients. (L, M) Calibration curves validating the predictive accuracy of nomogram for glioma patients’ survival of 1, 3, and 5years.

The prognostic value of MBOAT1 was assessed in TCGA and CGGA cohorts. Kaplan–Meier curve analysis showed that high MBOAT1 expression was significantly associated with shorter OS in all cohorts (Figure 2A–C). Both univariate Cox regression and multivariate Cox regression analyses identified MBOAT1 as an independent prognostic factor for OS (Figure 2D–G; Figure S4A, B). Subsequently, the nomogram model indicated that MBOAT1 was a good predictor of patient prognosis at 1, 3, and 5 years (Figure 2H,I; Figure S4C) The calibration curve confirmed satisfactory performance of our prognostic model (Figure 2J–M) (Figure S4D, E). Collectively, these results suggest its potential as a biomarker for glioma.

### 3.3 Mutation and Copy Number Variation Analysis

**FIGURE 3:**
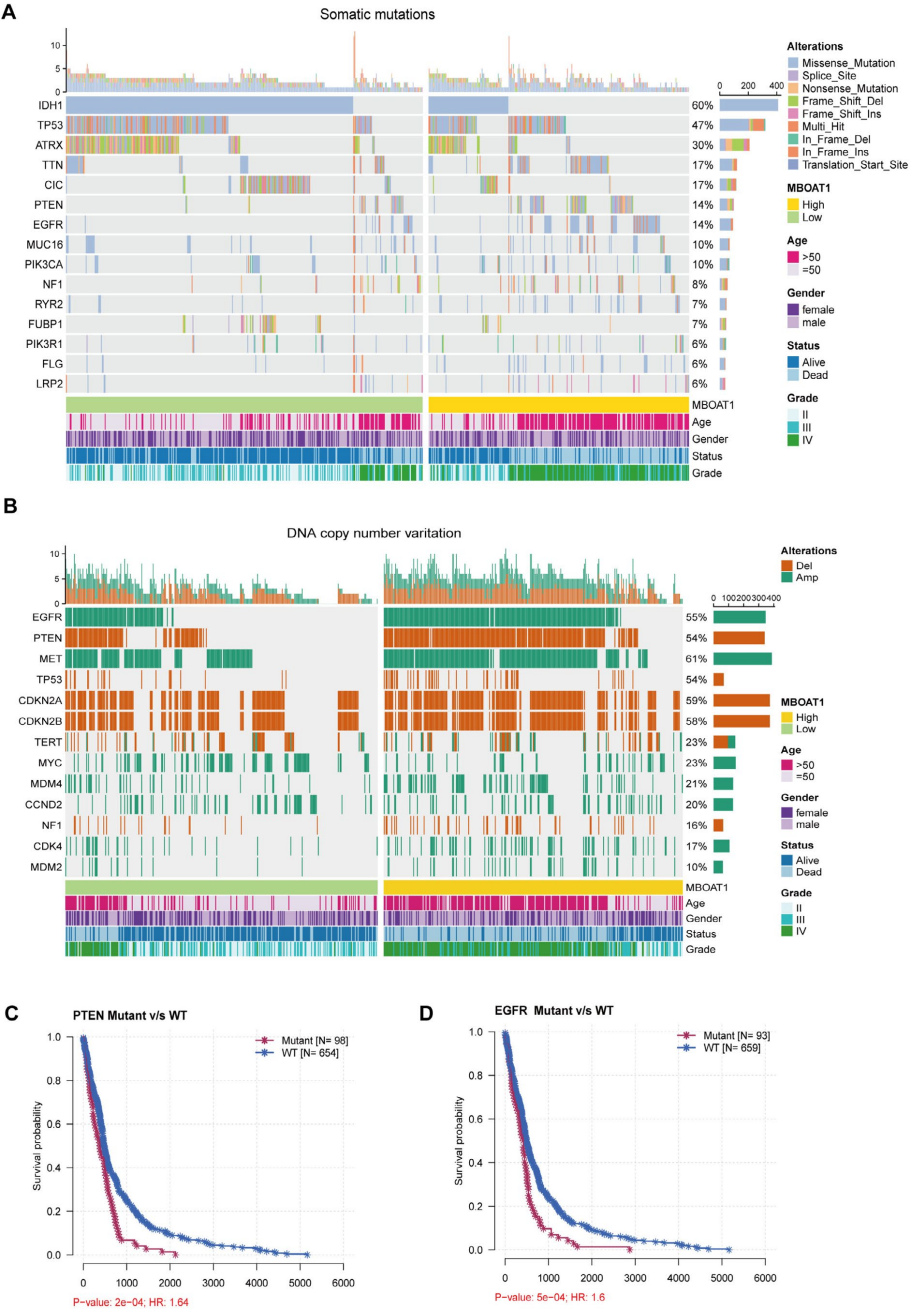
Mutation and copy number variation in TCGA cohort related to MBOAT1 expression. (A) Mutation landscape of key genes stratified by high and low MBOAT1 expression in glioma. (B) Copy number variation and their relationship to MBOAT1 expression. (C, D) Kaplan–Meier analyses of OS in glioma patients with PTEN and EGFR mutation in TCGA cohort.

To investigate the somatic mutation and copy number variation with MBOAT1 expression, we analyzed somatic mutation and copy number variation (CNV) data from the TCGA glioma cohort stratified by MBOAT1 expression levels. Elevated MBOAT1 expression was significantly associated with several molecular drivers of glioma pathogenesis, including IDH wild‐type status, EGFR amplification, PTEN deletion, MET amplification, TP53 loss, and CDKN2A/B homozygous deletion. These genomic alterations were also enriched in established high‐risk clinical subgroups, such as patients over 50 years of age and those with high‐grade tumors (Figure 3A,B). Survival analysis further confirmed that PTEN deletion and EGFR amplification, both linked to high MBOAT1 expression, were correlated with significantly reduced overall survival (Figure 3C,D). These results collectively suggest that MBOAT1 upregulation is related to a distinct genomic profile that contributes to malignant progression and poor clinical outcomes in glioma.

### 3.4 Functional Enrichment Shows MBOAT1 Related to Ferroptosis and TME

**FIGURE 4:**
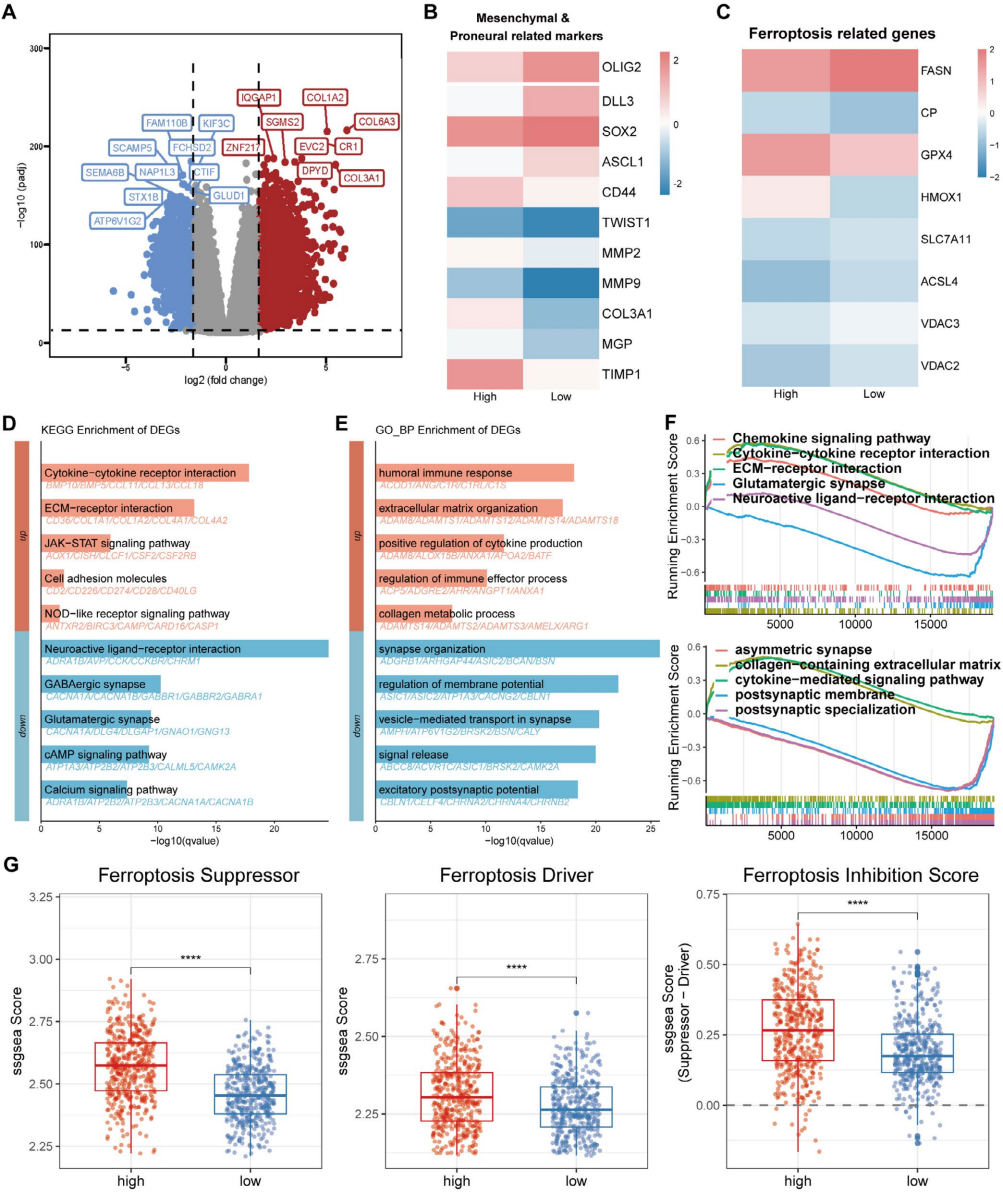
Functional enrichment of differential expression genes related to MBOAT1. (A) Volcano plot displays the differentially expressed genes by high and low MBOAT1 expression. (B, C) Heatmap shows mesenchymal, proneuronal, and ferroptosis marker genes in high and low MBOAT1 group in TCGA cohort. (D–E) The GOBP and KEGG enrichment analyses in TCGA cohort. (F) GSEA for TCGA cohort stratified by MBOAT1 expression levels. (G) ssGSEA for ferroptosis inhibition score related to MBOAT1 expression. Data are presented as mean ± SD. *p < 0.05; **p < 0.01; ***p < 0.001; ****p < 0.0001.

To explore the functional role of MBOAT1 in glioma, we performed differential expression analysis on TCGA samples stratified by high versus low MBOAT1 expression. We observed significant upregulation of COL1A2, COL6A3, and COL3A1 in the high‐expression group (Figure 4A), which are known to contribute to extracellular matrix (ECM) formation and enhance tumor migration and invasion. Further examination of marker genes for mesenchymal, proneuronal, and ferroptosis states demonstrated strong positive correlations between high MBOAT1 expression and both mesenchymal and ferroptosis signatures (Figure 4B,C). KEGG and GO enrichment analyses further demonstrated that differentially expressed genes (DEGs) in the high‐expression group were enriched in immune response and ECM‐related pathways, while DEGs in the low‐expression group showed enrichment in neural activity and ion channel‐related pathways (Figure 4D,E). These findings were corroborated by GSEA Analysis (Figure 4F). Then ssGSEA was performed based on ferroptosis suppressor and driver gene sets obtained from the FerrDb database [32]. Results indicated a significantly enhanced ferroptosis resistance phenotype in the MBOAT1 high‐expression group compared to the low‐expression group (Figure 4G).

**FIGURE 5:**
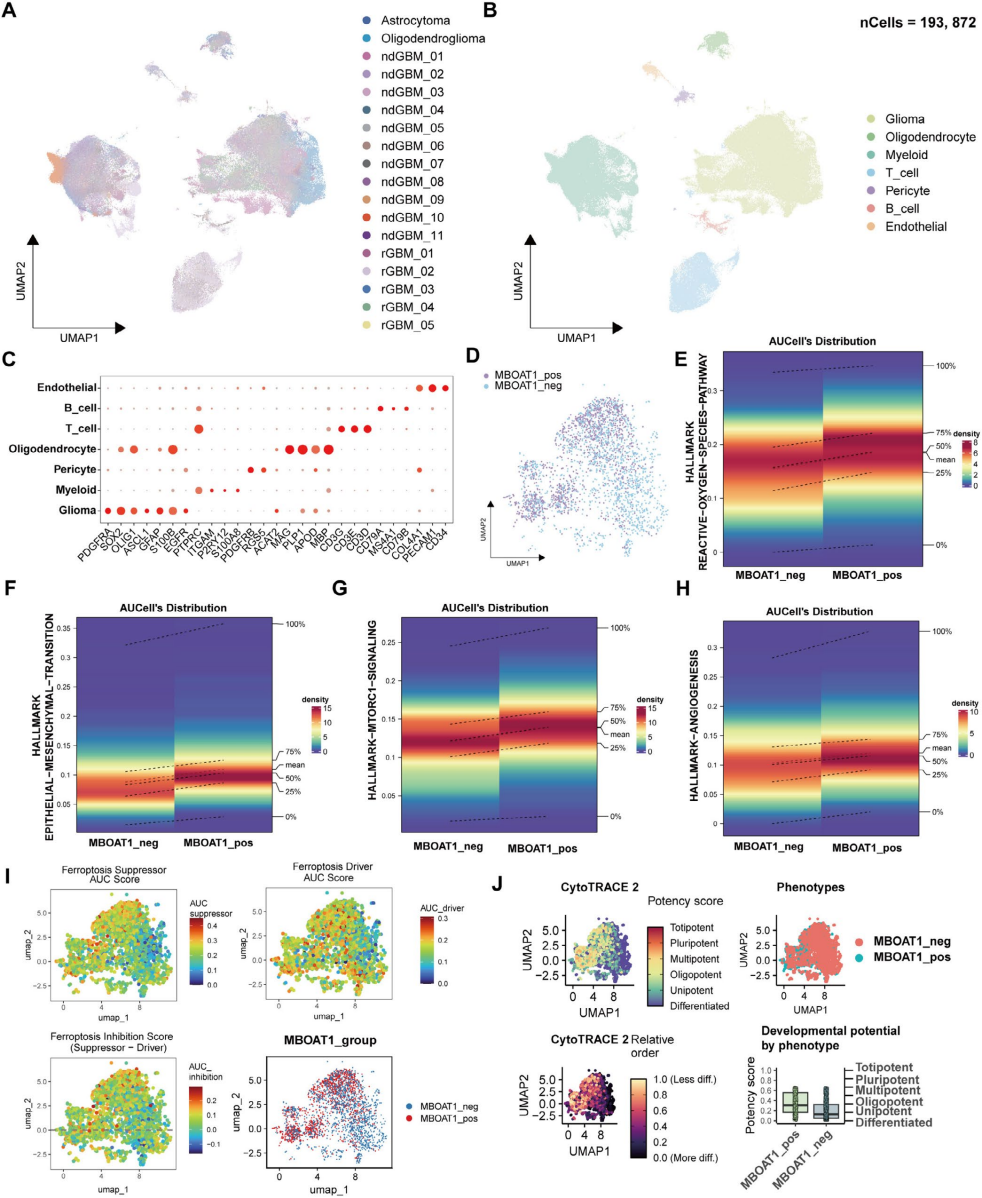
Single‐cell RNA‐seq analysis reveals that MBOAT1 drives glioma cells malignant progression and ferroptosis resistance. (A, B) The UMAP projections of 193,872 single cells from 18 patients showing the composition of different cell types in human gliomas. (C) Dot plot displays the expression patterns of all marker genes for glioma, myeloid, pericyte, oligodendrocyte, T cell, B cell, endothelial. (D) The UMAP The UMAP projections of 1355 MBOAT1‐positive glioma cells and 1355 MBOAT1‐negative glioma cells. (E–I) AUCell scoring differences between positive and negative MBOAT1 glioma cells. (J) Stemness properties analysis of positive and negative MBOAT1 glioma cells by CytoTRACE2.

To further investigate the heterogeneity of the glioma cells related to MBOAT1, we constructed a comprehensive glioma map based on single‐cell data from GEO; this cohort included 11 with newly diagnosed GBMs (ndGBM), 5 with recurrent GBMs (rdGBM), 1 with Astrocytoma, and 1 with Oligodendroglioma (Table S1). After filtering low‐quality cells and correcting the batch effect (Figure S5A), we obtained a total of 193,872 qualified cells (Figure S5B). Cluster analysis distinguished seven cell types: glioma (PDGFRA, SOX2, OLIG2, ASCL1, GFAP, S100B, EGFR), myeloid (P2RY12, ITGAM, S100A8), pericyte (PDGFRB, RGS5, ACAT2), oligodendrocyte (MAG, PLP1, APOD, MBP), T cell (CD3D, CD3G, CD3E), B cell (CD79A, CD79B, MS4A1), endothelial (COL4A1, PECAM1, CD34) (Figure 5A–C). Based on the MBOAT1 expression level, we extracted 1355 MBOAT1‐positive glioma cells and 1355 MBOAT1‐negative glioma cells for subsequent analysis (Figure 5D). Differential analysis was performed on two groups; among the top ten elevated genes in the positive cell population, NEAT1 significantly promotes glioma proliferation and migration, while EMP3 and ANXA1 facilitate the immunosuppressive tumor microenvironment [33, 34], MGST1 is a repressor of ferroptosis [35] and IFITM3 [36] enhances angiogenesis (Figure S6A). AUCell scoring of Hallmark pathways demonstrated significant upregulation of ROS, mTOR signaling, angiogenesis, DNA repair, epithelial‐mesenchymal transition (EMT), and TGF‐β pathways in MBOAT1‐positive cells (Figure 5E–H; Figure S6B–D). Moreover, ferroptosis suppressor genes showed notably enrichment in the MBOAT1 positive cluster (Figure 5I). Further assessment with CytoTRACE2 indicated substantially greater stemness potential in positive cells (Figure 5J). Collectively, these results indicate that the MBOAT1 positive cell population exhibits enhanced proliferation potential, invasive capacity, stemness properties, ferroptosis resistance, and immunosuppressive capabilities.

### 3.5 MBOAT1 Is Related to an Immunosuppressive Microenvironment

**FIGURE 6:**
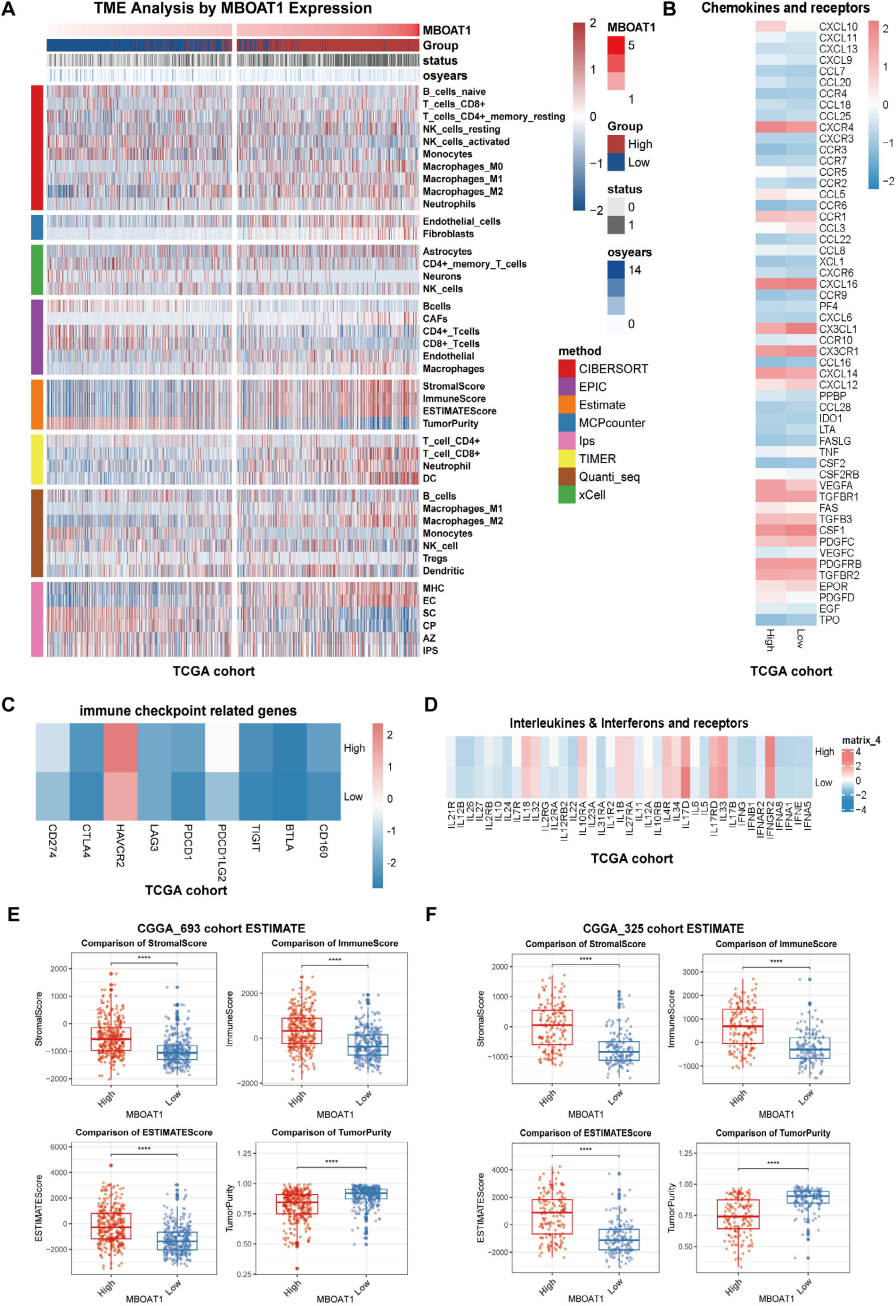
MBOAT1 regulates tumor infiltration of immune cells in TCGA and CGGA cohorts. (A) The heatmap shows the frequency of TME infltrating cells and immune score between high and low MBOAT1 groups in TCGA cohort. (B–D) The heatmap shows variations in mRNA expression of chemokines, interleukins, other cytokines and immune check points related genes between high and low MBOAT1 groups in TCGA cohort. (E, F) StromalScores, ImmuneSocres and Estimatescores comparison between high and low‐MBOAT1 expression groups in CGGA cohorts. Data are presented as mean ± SD. *p < 0.05; **p < 0.01; ***p < 0.001; ****p < 0.0001.

To determine whether MBOAT1 altered tumor microenvironment leads to poor prognosis in glioma, we performed integrated analysis using eight methods in TCGA cohort. This comprehensive profiling revealed that elevated MBOAT1 expression correlates with increased infiltration of M2 macrophages, fibroblasts, epithelial cells, astrocytes, cancer‐associated fibroblasts (CAFs), neutrophils, and dendritic cells (DCs). In contrast, low MBOAT1 expression associates with monocytes, CD4^+^ T cells, CD8^+^ T cells, neuronal cells, B cells, and NK cells. Also, ESTIMATE scores demonstrated significantly higher stromal and immune scores in high MBOAT1 group, indicating an immunosuppressive tumor microenvironment characterized by MBOAT1 expression (Figure 6A). Furthermore, transcriptional dysregulation was observed in interferon signaling, chemokine networks, and immune checkpoint genes between MBOAT1‐high and ‐low groups (Figure 6B–D). Two CGGA cohorts (CGGA_325, CGGA_693) also showed similar results using ESTIMATE and CIBERSORT (Figure 6E,F; Figure S7A–H).

**FIGURE 7:**
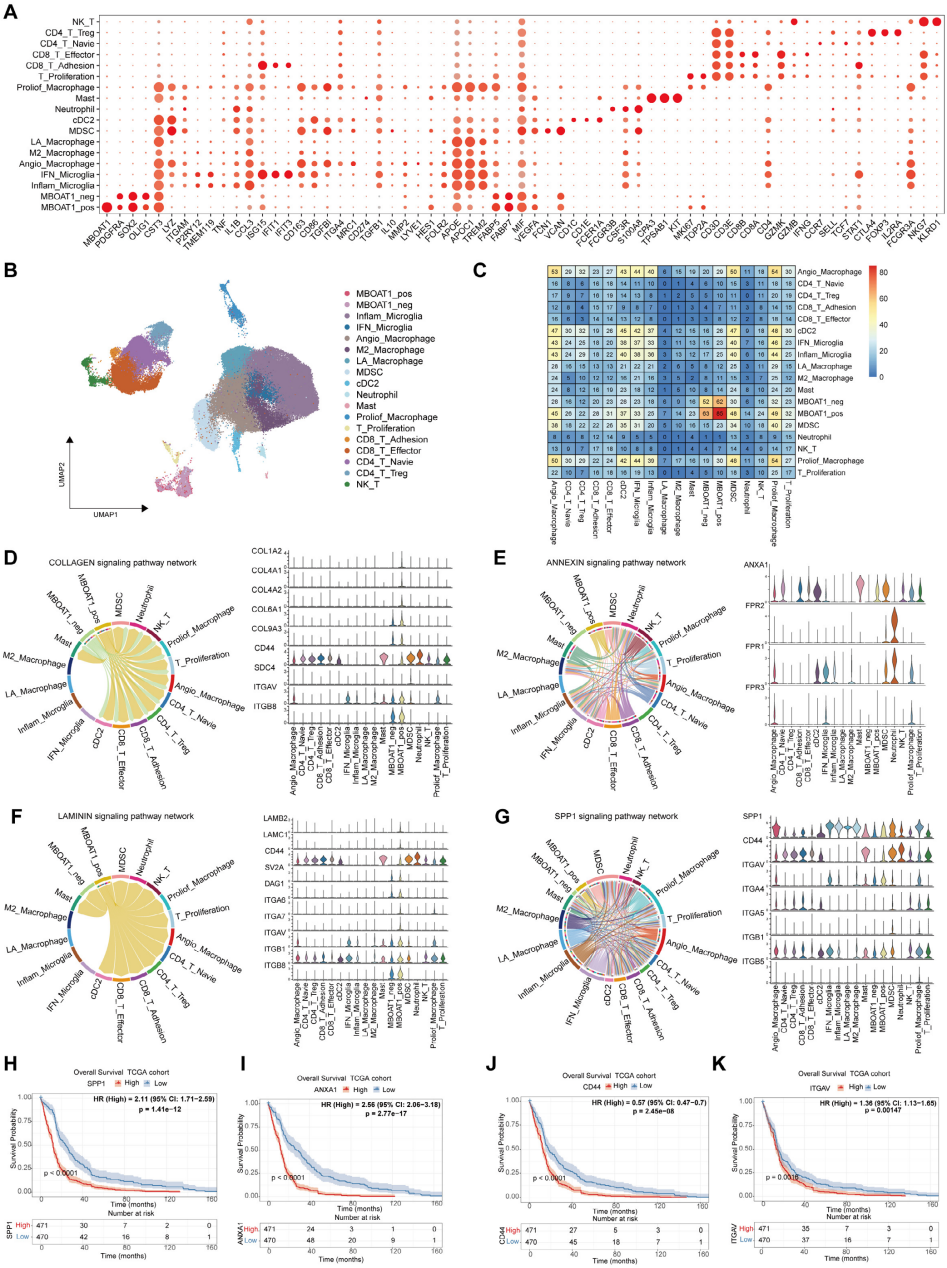
Single‐cell RNA‐seq analysis reveals that MBOAT1 promotes an immunosuppressive microenvironment. (A) Dot plot of marker genes for T cell and myeloid cell types. (B) The UMAP projections of 22,127 T cells and 68,693 myeloid cells from 18 glioma patients. (C) Heatmap displays the interactions between all immune cell types and MBOAT1 related glioma cell types. (D–G) Ligand‐receptor pairs difference between MBOAT1 related glioma cells and immune cell types. (H–K) Kaplan–Meier analyses of OS related to SPP1, ANXA1, CD44, ITGAV in glioma patients from the TCGA cohort.

To further explore immune infiltrate heterogeneity associated with MBOAT1, we extracted immune cell clusters and further subdivided 22,127 T cells and 68,693 myeloid cells into subpopulations, annotated by specific marker genes: Myeloid (CST3, LYZ, ITGAM), Microglia (P2RY12, TMEM119), Macrophage (TGFBI, ITGA4), Inflam_Microglia (TNF, IL1B, CCL3), IFN_Microglia (ISG15, IFIT1, IFIT3), Angio_Macrophage (LYVE1, HES1, FOLR2), M2_Macrophage (CD163, CD68), LA_Macrophage (APOE, APOC1, TREM2, FABP5, FABP7), MDSC (MIF, VEGFA, FCN1, VCAN), cDC2 (CD1C FCER1A, FCER1A), Mast (CPA3, TPSAB1, KIT), Neutrophil (FCGR3B, CSF3R, S100A8), Proliof_Macrophage (MKI67, TOP2A),T cell (CD3D, CD3E, CD3G), CD8_T cell (CD8A, CD8B), CD4_T cell (CD4A), T_Proliferation (MKI67, TOP2A), CD8_T_Adhesion (STAT1, IFIT1, IFIT3), CD8_T_Effector (GZMK, GZMB, IFNG), CD4_T_Navie (CCR7, SELL, TCF7), CD4_T_Treg (CTLA4, FOXP3, IL2RA), NK_T (FCGR3A, NKG7, KLRD1) (Figure 7A). Then we integrated these immune cell subpopulations and MBOAT1 related cell types for subsequent analysis (Figure 7B). CellChat was employed to predict the ligand‐receptor interactions between two MBOAT1 subclusters and other immune cell types; there are more interaction numbers between MBOAT1‐positive glioma cells and immune cell types compared to the MBOAT1‐negative group (Figure 7C). The COLLAGEN, LAMININ, ANNEXIN, SPP1, ApoE, CD99, Cholesterol, FN1 signaling networks exhibited stronger interactions between MBOAT1‐positive glioma cells and immune cell types (Figure 7D–G; Figure S8A–D). Survival analysis revealed that high expression of SPP1, ANXA1, CD44 and ITGAV was significantly associated with shorter overall survival in glioma patients (Figure 7H–K). These results suggest that MBOAT1 may promote the polarization of macrophages, microglia and T cell subtypes in the tumor microenvironment into immunosuppressive phenotypes.

### 3.6 Mboat1 Promotes GBM Cell Proliferation, Migration and Invasion

**FIGURE 8:**
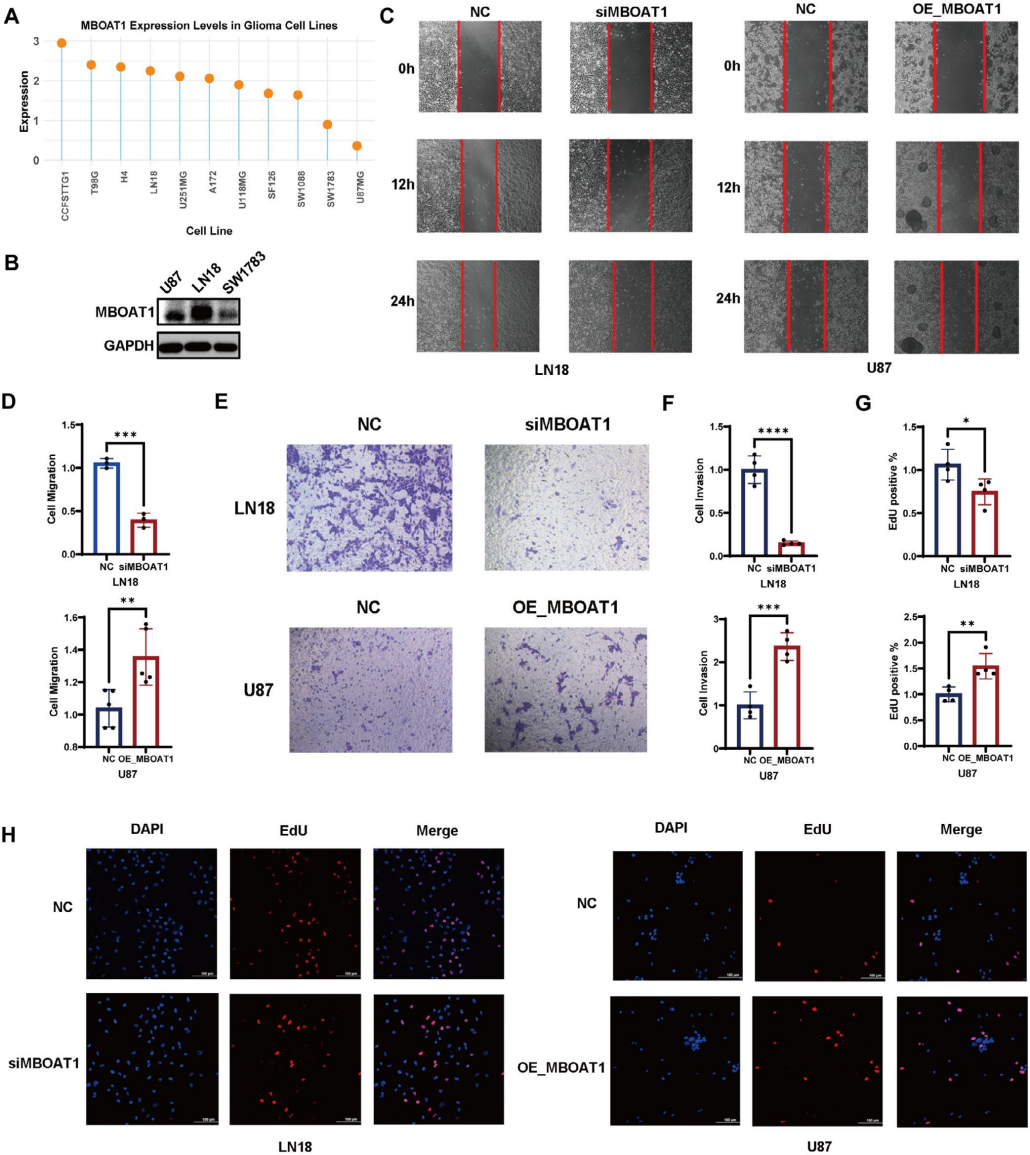
MBOAT1 promotes GBM cell proliferation, migration and invasion. (A) MBOAT1 mRNA expression across 11 glioma cell lines in the CCLE database. (B) Western blot of MBOAT1 protein in LN18 and U87 cells. (C, D) Wound healing assay, quantified by the 24‐h average migration distance, showed that MBOAT1 knockdown inhibited LN18 cell migration by 60%, while its overexpression promoted U87 cell migration by 30%. (E, F) Transwell assay demonstrated that MBOAT1 knockdown suppressed invasion in LN18 and overexpression of MBOAT1 promoted invasion in U87 cells. (G, H) EdU assay to measure cell proliferation in LN18 and U87 after knockdown and overexpression of MBOAT1. Data are presented as mean ± SD. *p < 0.05; **p < 0.01; ***p < 0.001; ****p < 0.0001.

To investigate the impact of MBOAT1 on the biological functions of GBM cells, we first analyzed MBOAT1 expression across 11 glioma cell lines in the CCLE database. Based on this screening, we selected GBM cell line: U87 with low MBOAT1 expression and LN18 with high expression MBAOT1 (Figure 8A,B). After validating the transfection efficiency and confirming that MBOAT1 mRNA levels were effectively altered by siRNA knockdown and plasmid overexpression in vitro (Figure S9A), functional assays. Transwell and scratch wound healing assays demonstrated that MBOAT1 overexpression significantly enhanced the invasion and migration capabilities of U87MG cells. Conversely, MBOAT1 knockdown markedly suppressed the migratory and invasive abilities of LN18 cells (Figure 8C–F). Furthermore, EdU proliferation assays indicated that MBOAT1 also modulates the proliferative capacity of GBM cells (Figure 8G,H).

### 3.7 MBOAT1 Enhances the Ferroptosis Resistance in GBM Cells

**FIGURE 9:**
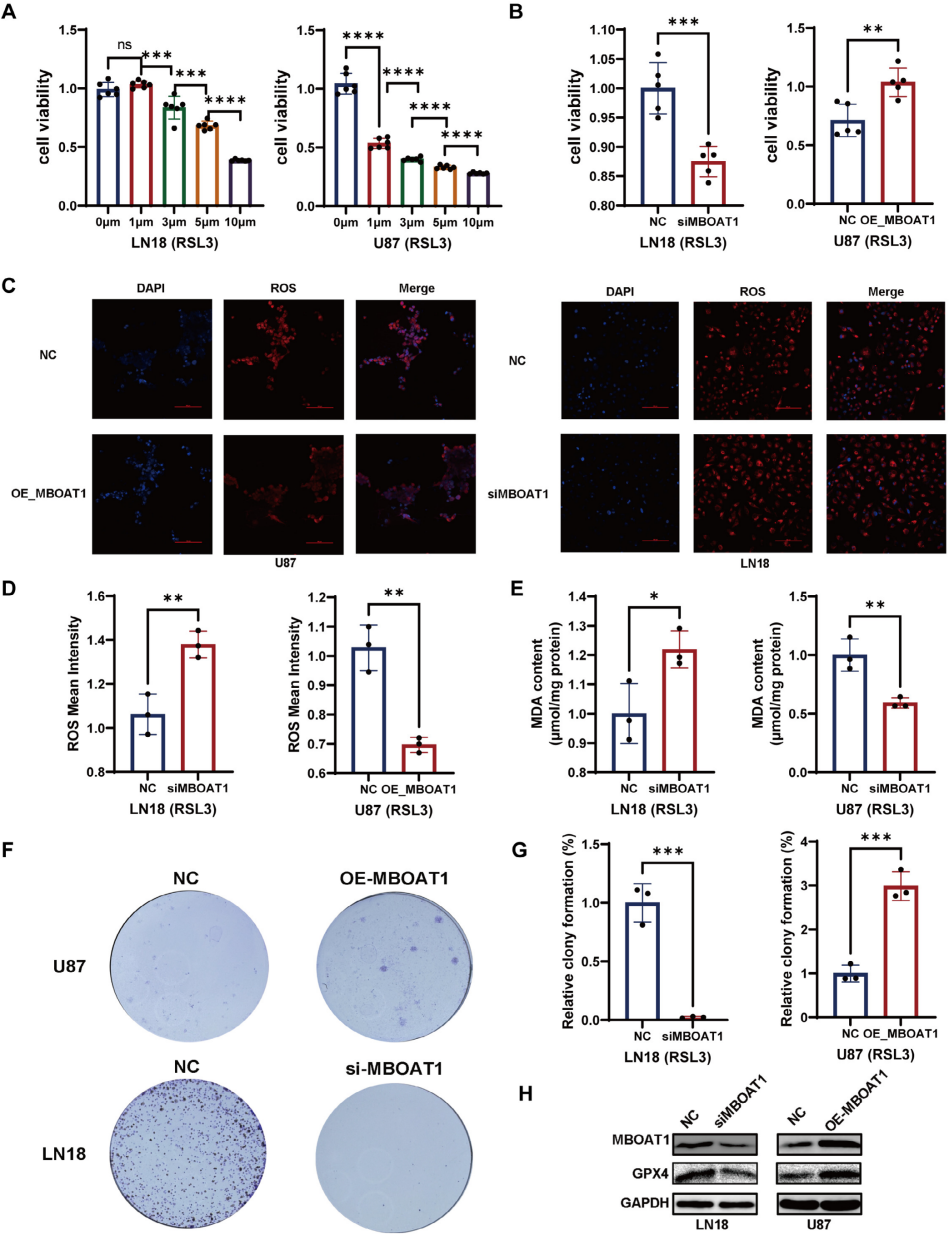
MBOAT1 enhances the ferroptosis resistance in GBM cells. (A) Cell viability of LN18 and U87 treated with RSL3 (0, 1, 3, 5, 10 μM). (B) CCK‐8 assay to measure cell viability after MBOAT1 knockdown in LN18 and overexpression in U87 cells. (C, D) ROS detection after MBOAT1 knock down in LN18 and overexpression of MBOAT1 in U87 cells. (E) MDA assay showed MBOAT1 knockdown increased the lipid peroxidation in LN18 and overexpression of MBOAT1 suppressed the process of lipid peroxidation in U87 cells. (F, G) Colony formation assay of LN18 and U87 after knock down or overexpression of MBOAT1. (H) Western blot of MBOAT1 and GPX4 in LN18 or U87 cells treated with RSL3 after transfection with siRNA or plasmid. Data are presented as mean ± SD. *p < 0.05; **p < 0.01; ***p < 0.001; ****p < 0.0001.

To explore whether MBOAT1 promotes glioma cell survival by enhancing ferroptosis resistance, we first performed gradient concentration screening of the ferroptosis inducer RSL3. CCK‐8 assays showed that LN18 cells displayed higher viability than U87MG cells at the same RSL3 concentrations (Figure 9A). Based on these results, we selected 5 μM RSL3 for LN18 treatment and 1 μM RSL3 for U87MG treatment. Following MBOAT1 overexpression in U87MG and knockdown in LN18, we then assessed the cell growth inhibition rates resulting from treatments with RSL3; significantly increased viability in MBOAT1‐overexpressing U87MG and markedly decreased viability in MBOAT1‐knockdown LN18 were observed (Figure 9B). We next measured ROS levels after RSL3 treatment. Overexpression of MBOAT1 in U87MG cells led to a decrease in ROS, while knockdown of MBOAT1 in LN18 cells resulted in elevated ROS (Figure 9C,D). Consistent with these findings, MDA assays revealed that MBOAT1 knockdown significantly increased lipid peroxidation after RSL3 treatment, whereas MBOAT1 overexpression suppressed it (Figure 9E). Colony formation assays further demonstrated that MBOAT1 enhanced survival under ferroptosis stress: overexpression in U87MG promoted colony formation, and knockdown in LN18 impaired it (Figure 9F,G). Western blot further confirmed that ferroptosis marker protein GPX4 had the same trend with MBOAT1 (Figure 9H). These results suggested MBOAT1 promotes ferroptosis resistance in vitro.

### 3.8 MBOAT1 Promotes Ferroptosis Resistance and Is Associated With Immunosuppressive Microenvironment in Nude Mice

**FIGURE 10:**
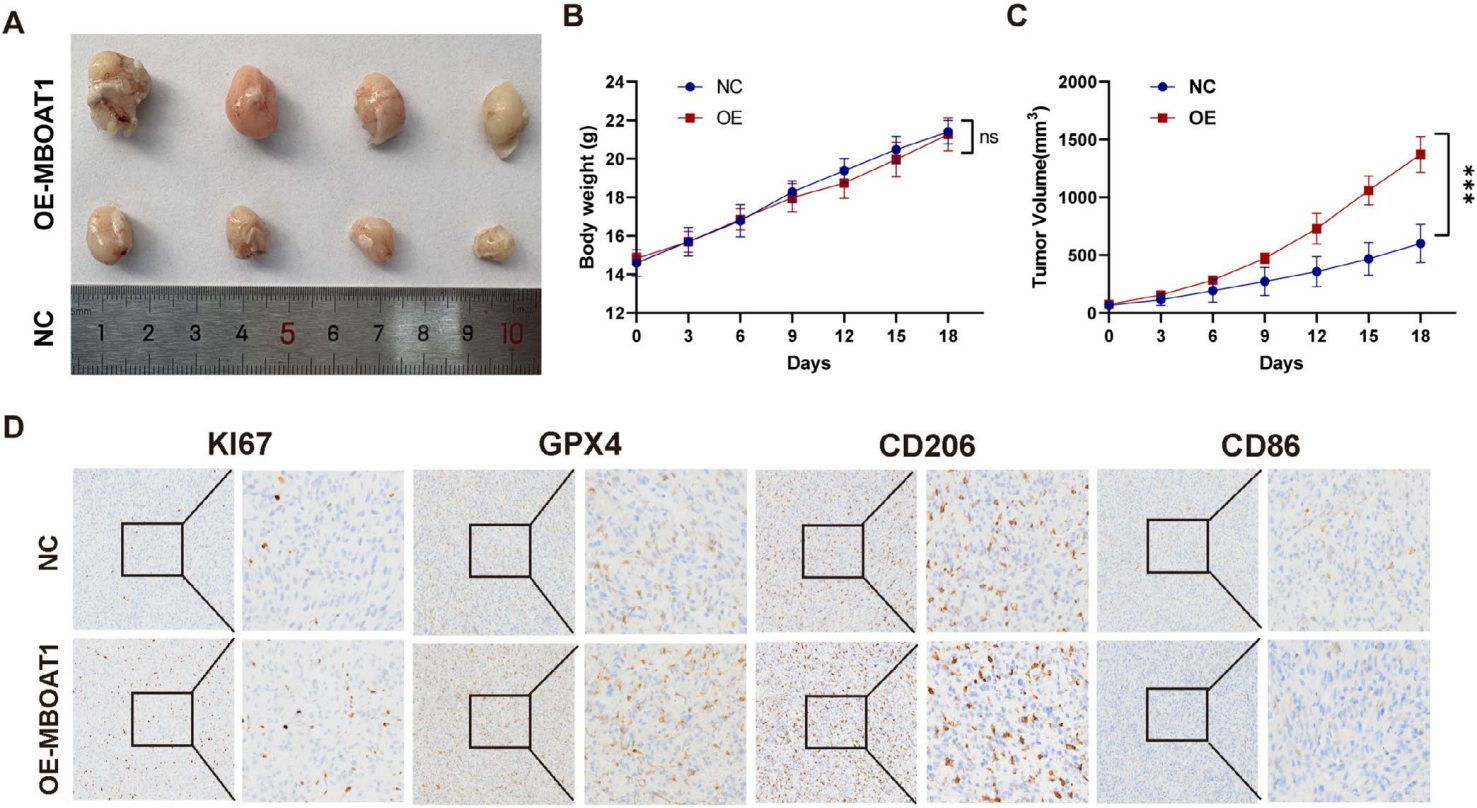
MBOAT1 promotes ferroptosis resistance and immunosuppressive microenvironment in nude mice. (A) Tumor growth of mice implanted subcutaneously with U87 cells. (B, C) Tumor volume and body weight were measured. (D) Immunohistochemistry revealed the expression of GPX4, CD206, CD86 and Ki67 of tumors in mice. Data are mean ± SD, *n* = 4 mice per group. *p < 0.05; **p < 0.01; ***p < 0.001; ****p < 0.0001.

In the subcutaneous xenograft, nude mice received intraperitoneal administration of RSL3 (3 mg/kg) 10 days after tumor implantation. Tumors derived from MBOAT1‐overexpressing U87MG cells displayed significantly accelerated growth compared to those in control groups (Figure 10A–C). Immunohistochemical analysis further revealed a significant upregulation of Ki67 and GPX4 expression in the MBOAT1‐overexpressing group. Additionally, the expression of the M2 macrophage marker CD206 was upregulated, concomitantly with a downregulation of the M1 marker CD86, which is correlated with a shift in macrophage polarization within the tumor microenvironment (Figure 10D).

## 4 Discussion

Ferroptosis is recognized as programmed cell death which is characterized by lipid peroxidation. Recent studies proposed that ferroptosis is widely involved in tumor progression and anticancer immunity [37]. In this study, we comprehensively investigated the role of MBOAT1 in glioma using transcriptomic and single‐cell RNA sequencing data from TCGA, CGGA, and GEO databases. Our analyses revealed that MBOAT1 is significantly upregulated in glioma tissues compared to non‐tumor brain tissues, and its expression positively correlated with higher grades of glioma and established markers of poor prognosis, including older age, non‐1p/19q codeletion, IDH wild‐type status, and unmethylated MGMT promoter. Kaplan–Meier method, ROC curves, univariate Cox regression, and the nomogram consistently indicated patients with higher expression of MBOAT1 have shorter overall survival time. Somatic mutation and DNA copy number analysis also showed that MBOAT1 has a strong relationship with EGFR, PTEN and other mutations that are associated with aggressive tumor behavior. Therefore, we identified MBOAT1 as a novel potential biomarker for the prognosis in glioma.

To elucidate the mechanisms underlying MBOAT1's role, we performed functional enrichment analyses (GO, KEGG, and GSEA). The results revealed significant associations with ECM organization, inflammation and immune‐related pathways. ssGSEA further indicated enhanced ferroptosis resistance in MBOAT1‐high tumor cells. Single‐cell analysis using AUCell scoring also revealed that the MBOAT1‐positive glioma cell population exhibited enhanced capabilities in angiogenesis, epithelial mesenchymal transition, DNA repair, mTOR signaling, and ROS‐related pathways. Although ROS released during inflammatory processes can induce ferroptosis [38], pathways such as mTOR and DNA repair significantly promote tumor cell survival. Consequently, MBOAT1‐positive cells may possess a higher survival capacity even in environments with elevated inflammation or ROS levels. Subsequent ferroptosis‐related gene set scoring analysis of single‐cell transcriptomes confirmed that MBOAT1‐positive glioma cells indeed have a greater resistance to ferroptosis.

We observed a link between high MBOAT1 expression and M2 macrophage infiltration, suggesting its potential association with an immunosuppressive microenvironment. This was further supported by single‐cell RNA sequencing analysis, which indicated enhanced communication between MBOAT1‐high glioma cells and immune cells, mediated by upregulated signaling pathways involving collagen, annexin, laminin, and integrin families. Furthermore, our CytoTRACE2 analysis indicates that the MBOAT1‐positive glioma cell population exhibits higher stemness and differentiation potential. This finding prompts an examination of its potential link to glioma stem cells (GSCs), which are established master regulators of tumor progression and the immunosuppressive TME [39, 40, 41]. Critically, recent mechanistic studies have elucidated how specific molecules enriched in GSCs, such as ARPC1B and MIR222HG, actively bridge stem cell properties with immune evasion. ARPC1B has been shown to maintain the mesenchymal, therapy‐resistant phenotype of GSCs while contributing to microenvironment modulation [39, 42, 43]. Similarly, MIR222HG drives the proneural‐to‐mesenchymal transition in GSCs and concurrently promotes the immunosuppressive polarization of macrophages [44]. Therefore, building on this established concept and our data, we hypothesize that the observed association between MBOAT1‐high glioma cells and an altered immune landscape may be partly attributable to their acquired stem‐like properties, which could enable them to similarly foster an immunosuppressive TME.

Functional validation in vitro demonstrated that MBOAT1 knockdown significantly suppressed glioma cell proliferation, migration, and invasion. Under treatment with the ferroptosis inducer RSL3, MBOAT1 depletion sensitized cells to ferroptosis, as indicated by elevated lipid ROS and MDA levels, alongside reduced cell viability. These findings were corroborated in a xenograft model, where MBOAT1‐overexpressing tumors exhibited accelerated growth and increased ferroptosis resistance. IHC analysis further revealed elevated M2 macrophage infiltration in MBOAT1‐high tumors. To establish causality, future studies will employ functional assays such as co‐culture systems and orthotopic tumor models in immunocompetent C57BL/6 mice to determine whether MBOAT1 actively drives macrophage recruitment and polarization. Specifically, it will be crucial to investigate whether potential ligand‐receptor axes identified in our single‐cell analysis, such as SPP1‐CD44/ITGAV, serve as key mechanistic links through which MBOAT1‐high tumor cells communicate with and polarize macrophages.

Taken together, our results support that MBOAT1 enhances tumor growth, at least in part, by conferring ferroptosis resistance. However, while our data link MBOAT1 to this phenotype using established secondary markers, they do not directly validate the molecular mechanism by which MBOAT1 alters membrane phospholipid composition to suppress peroxidation. Targeted lipidomic profiling will be essential to quantify changes in phospholipid species, such as PE‐MUFA versus PE‐PUFA ratios, upon MBOAT1 modulation. Future work should investigate whether MBOAT1 contributes to the immunosuppressive microenvironment by maintaining the stemness of glioma stem cells or by influencing macrophage polarization. Furthermore, to deepen the mechanistic understanding, investigation should extend upstream to explore how MBOAT1 expression or activity is regulated within the broader lipid metabolic network. This includes examining potential alterations in key pathways such as de novo fatty acid synthesis, which may supply substrates for MBOAT1.

In conclusion, our investigation identified MBOAT1 as an oncogenic driver in glioma that promotes ferroptosis resistance and is associated with immunosuppressive TME. These findings highlight its prognostic value and suggest that targeting MBOAT1 may represent a promising therapeutic strategy. Direct mechanistic validation through lipidomic profiling and immunocompetent orthotopic models will be critical to fully elucidate its role and translational potential.

## Author Contributions

Junqi Fan analyzed data and conducted the experiments, and contributed to writing – review and editing. Qingqing Huang contributed to writing – review and editing and data curation. Lanxin Bao conducted the experiments. Xueran Chen contributed to supervision, writing – original draft, and visualization. Zhiyou Fang contributed to supervision, writing – original draft, and funding acquisition. Haifeng Shu contributed to supervision, writing – original draft, and funding acquisition. All authors have read and approved the final version of the manuscript.

## Funding

This work was supported by Sichuan Tianfu Qingcheng Plan Project [No. 1867], Hospital‐level Talent Nurturing Program [no number], the National Natural Science Foundation of China (grant numbers 82172663 and 32470775) and the Key Program of Natural Science Foundation of Sichuan Province (Grant No. 2026NSFSCZY0040).

## Ethics Statement

The study was approved by the Institutional Review Board of Hefei Cancer Hospital, Chinese Academy of Sciences (approval number SWYX‐Y‐2021‐41).

## Consent

The authors have nothing to report.

## Conflicts of Interest

The authors declare no conflicts of interest.

## Supporting information


**Figure S1:** MBOAT2/5/7 expression profiles in the database. (A), MBOAT2 (B), MBOAT5 (C), MBOAT7 expression levels in pan‐cancer from TCGA database. (D) Relative expression levels of MBOAT1 in glioma tissues and normal tissues from TCGA and GTEx database. Data are presented as mean ± SD. *p < 0.05; **p < 0.01; ***p < 0.001; ****p < 0.0001.
**Figure S2:** Correlation of MBOAT1 with clinical variables. (A) Gender differences of MBOAT1/2, ESR1 and AR expression in LGG and GBM. (B, C) Association between MBOAT1 and WHO grade in CGGA cohorts. Box plots showing the association between MBOAT1 and various clinical variables in CGGA_325 cohort, including gender (D), age (E), IDH mutation status (F), 1p19q codeletion status (G), MGMTp methylation status (H), primary and recurrent subtype (I). Data are presented as mean ± SD. *p < 0.05; **p < 0.01; ***p < 0.001; ****p < 0.0001.
**Figure S3:** Relationship between MBOAT1 and clinical variables. (A‐F) Boxplots of association between MBOAT1 and various clinical variables in CGGA_693 cohort. (G‐I) The association between MBOAT1 and histology in TCGA and CGGA cohorts. (J, K) The Sankey diagram demonstrates the relationships between clinical outcomes and several clinical features in CGGA cohorts. Data are presented as mean ± SD. *p < 0.05; **p < 0.01; ***p < 0.001; ****p < 0.0001.
**Figure S4:** The prognostic value of MBOAT1 expression in CGGA_693 cohort. (A) Univariate Cox regression analysis of clinical pathological characteristics in CGGA_693 cohort. (B) Multivariate Cox regression analysis of clinical pathological characteristics in CGGA_693 cohort. (C) A nomogram was utilized to predict the OS of glioma patients from the CGGA_693 cohort. (D) ROC curves to evaluate the predictive ability of MBOAT1 on survival time of patients in CGGA_693 cohort. (E) Calibration curves of the nomogram for predicting 1, 3, and 5 years in in glioma patients from CGGA_693 cohort.
**Figure S5:** Batch effect correct and clustering. (A) PCA plot of the batch effect before and after harmony. (B) The UMAP and tSNE map shows cell clusters in different patients. *p < 0.05; **p < 0.01; ***p < 0.001; ****p < 0.0001.
**Figure S6:** Differential analysis and AUCell scoring. (A) Volcano plot displays the differentially expressed genes in MBOPAT1 positive and negative groups. (B) Density plot shows differences in the TGF‐β pathway between two groups. ****p < 0.0001.
**Figure S7:** MBOAT1 regulates tumor infiltration of immune cells in CGGA cohorts. (A, B) The box plot shows the frequency of TME infltrating cells between high and low MBOAT1 groups in CGGA_325 and CGGA_693 cohorts. (C‐H) The heatmap shows variations in mRNA expression of chemokines, interleukins, other cytokines and immune check points related genes between high and low MBOAT1 groups in CGGA cohorts. *p < 0.05; **p < 0.01; ***p < 0.001; ****p < 0.0001.
**Figure S8:** Cellchat pathway analysis between positive and negative MBOAT1 glioma cells. (A‐D) Chord diagrams and violin plots demonstrate differential distribution of receptor‐ligand pairs.
**Figure S9:** Transfection efficiency assay. (A) Bar plots depict plasmid overexpression efficiency and siRNA knockdown efficiency in LN18 and U87 cells. Data are presented as mean ± SD. *p < 0.05; **p < 0.01; ***p < 0.001; ****p < 0.0001.

**Table S1:** Clinical information of the glioma patients.

## Data Availability

The data that support the findings of this study are available from The Cancer Genome Atlas (TCGA), the Chinese Glioma Genome Atlas (CGGA), and the Gene Expression Omnibus (GEO) of the National Center for Biotechnology Information at https://portal.gdc.cancer.gov, http://www.cgga.org.cn, and http://www.ncbi.nlm.nih.gov/geo/, respectively.
