# Supplementary material for: MBOAT1 Promotes Glioma Progression Through Enhancing Ferroptosis Resistance and Immunosuppressive Microenvironment
Source: CNS Neurosci Ther. 2026 Feb 16;32(2):e70785. doi: 10.1002/cns.70785 (PMC12908195; doi:10.1002/cns.70785)
Supplement: Supplementary file 1 — Figure S1: MBOAT2/5/7 expression profiles in the database. (A), MBOAT2 (B), MBOAT5 (C), MBOAT7 expression levels in pan‐cancer from TCGA database. (D) Relative expression levels of MBOAT1 in glioma tissues and normal tissues from TCGA and GTEx database. Data are presented as mean ± SD. *p < 0.05; **p < 0.01; ***p < 0.001; ****p < 0.0001. Figure S2: Correlation of MBOAT1 with clinical variables. (A) Gender differences of MBOAT1/2, ESR1 and AR expression in LGG and GBM. (B, C) Association between MBOAT1 and WHO grade in CGGA cohorts. Box plots showing the association between MBOAT1 and various clinical variables in CGGA_325 cohort, including gender (D), age (E), IDH mutation status (F), 1p19q codeletion status (G), MGMTp methylation status (H), primary and recurrent subtype (I). Data are presented as mean ± SD. *p < 0.05; **p < 0.01; ***p < 0.001; ****p < 0.0001. Figure S3: Relationship between MBOAT1 and clinical variables. (A‐F) Boxplots of association between MBOAT1 and various clinical variables in CGGA_693 cohort. (G‐I) The association between MBOAT1 and histology in TCGA and CGGA cohorts. (J, K) The Sankey diagram demonstrates the relationships between clinical outcomes and several clinical features in CGGA cohorts. Data are presented as mean ± SD. *p < 0.05; **p < 0.01; ***p < 0.001; ****p < 0.0001. Figure S4: The prognostic value of MBOAT1 expression in CGGA_693 cohort. (A) Univariate Cox regression analysis of clinical pathological characteristics in CGGA_693 cohort. (B) Multivariate Cox regression analysis of clinical pathological characteristics in CGGA_693 cohort. (C) A nomogram was utilized to predict the OS of glioma patients from the CGGA_693 cohort. (D) ROC curves to evaluate the predictive ability of MBOAT1 on survival time of patients in CGGA_693 cohort. (E) Calibration curves of the nomogram for predicting 1, 3, and 5 years in in glioma patients from CGGA_693 cohort. Figure S5: Batch effect correct and clustering. (A) PCA plot of the batch effect be [file CNS-32-e70785-s001.docx]

**MBOAT1 promotes glioma progression through enhancing ferroptosis resistance and immunosuppressive microenvironment**

**Junqi Fan^1^, Qingqing Huang^3^, Lanxin Bao^2^, Xueran Chen^2^*****, Zhiyou Fang^2^*****, Haifeng Shu^1^***

1 Department of Neurosurgery, The General Hospital of Western Theater Command, Chengdu, 610083, China.

2 Hefei Cancer Hospital of CAS; Institute of Health and Medical Technology, Hefei Institutes of Physical Science, Chinese Academy of Sciences (CAS), No. 350, Shushan Hu Road, Hefei 230031, Anhui, China.

3 Department of Anesthesiology, The General Hospital of Western Theater Command, Chengdu, 610083, China.

*Corresponding authors.[shuhaifeng@swjtu.edu.cn](mailto:shuhaifeng@swjtu.edu.cn); xueranchen@cmpt.ac.cn; [z.fang@cmpt.ac.cn](mailto:z.fang@cmpt.ac.cn)

Contributing authors: [1430979228@qq.com](mailto:1430979228@qq.com); [970327481@qq.com](mailto:970327481@qq.com); [baolanxin0929@163.com](mailto:baolanxin0929@163.com);


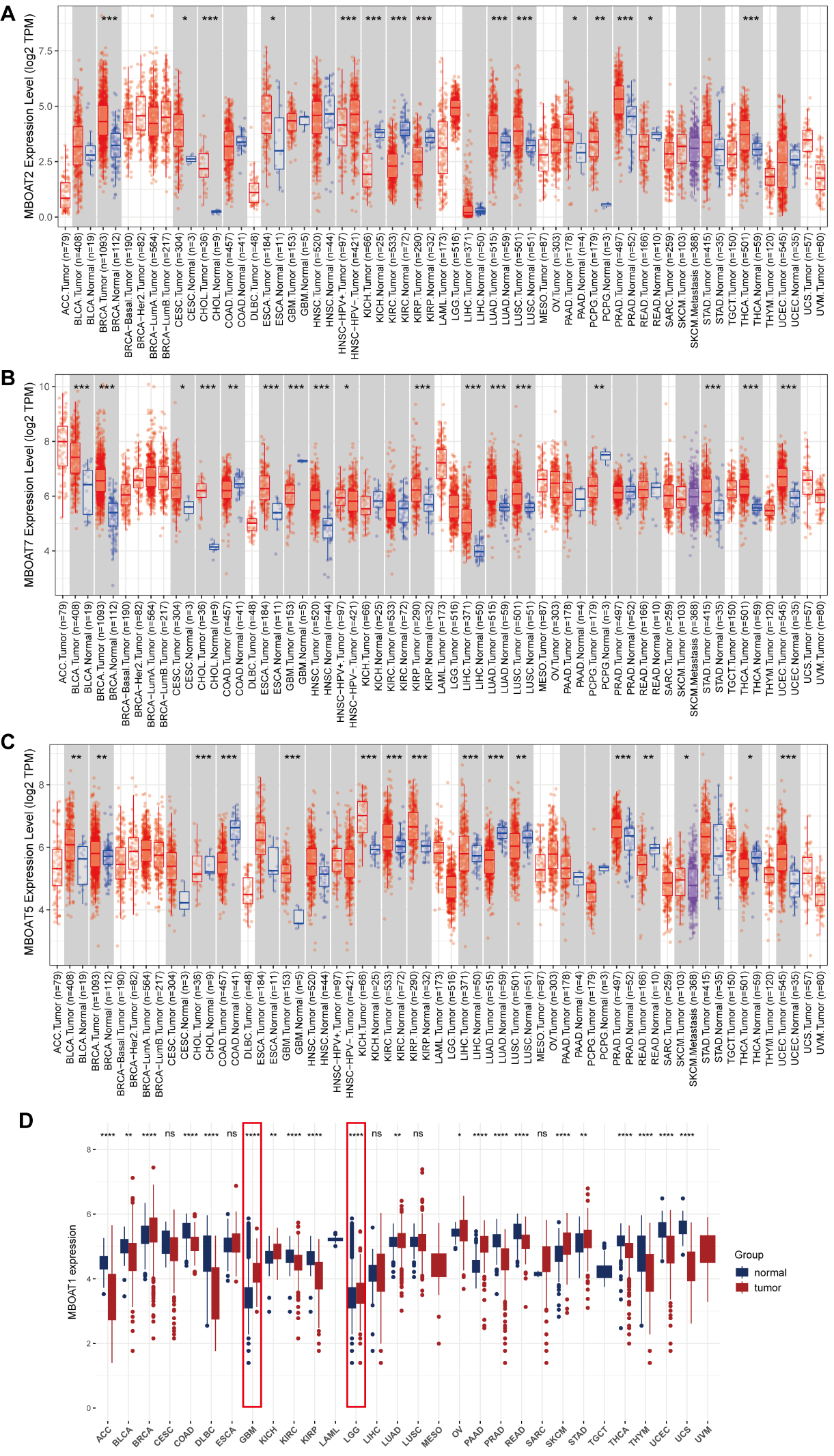
 Figure.S1 MBOAT2/5/7 expression profiles in the database. (**A**), MBOAT2 (**B**), MBOAT5 (**C**), MBOAT7 expression levels in pan-cancer from TCGA database. (**D**) Relative expression levels of MBOAT1 in glioma tissues and normal tissues from TCGA and GTEx database. Data are presented as mean ± SD. *P<0.05; **P<0.01; ***P<0.001; ****P<0.0001


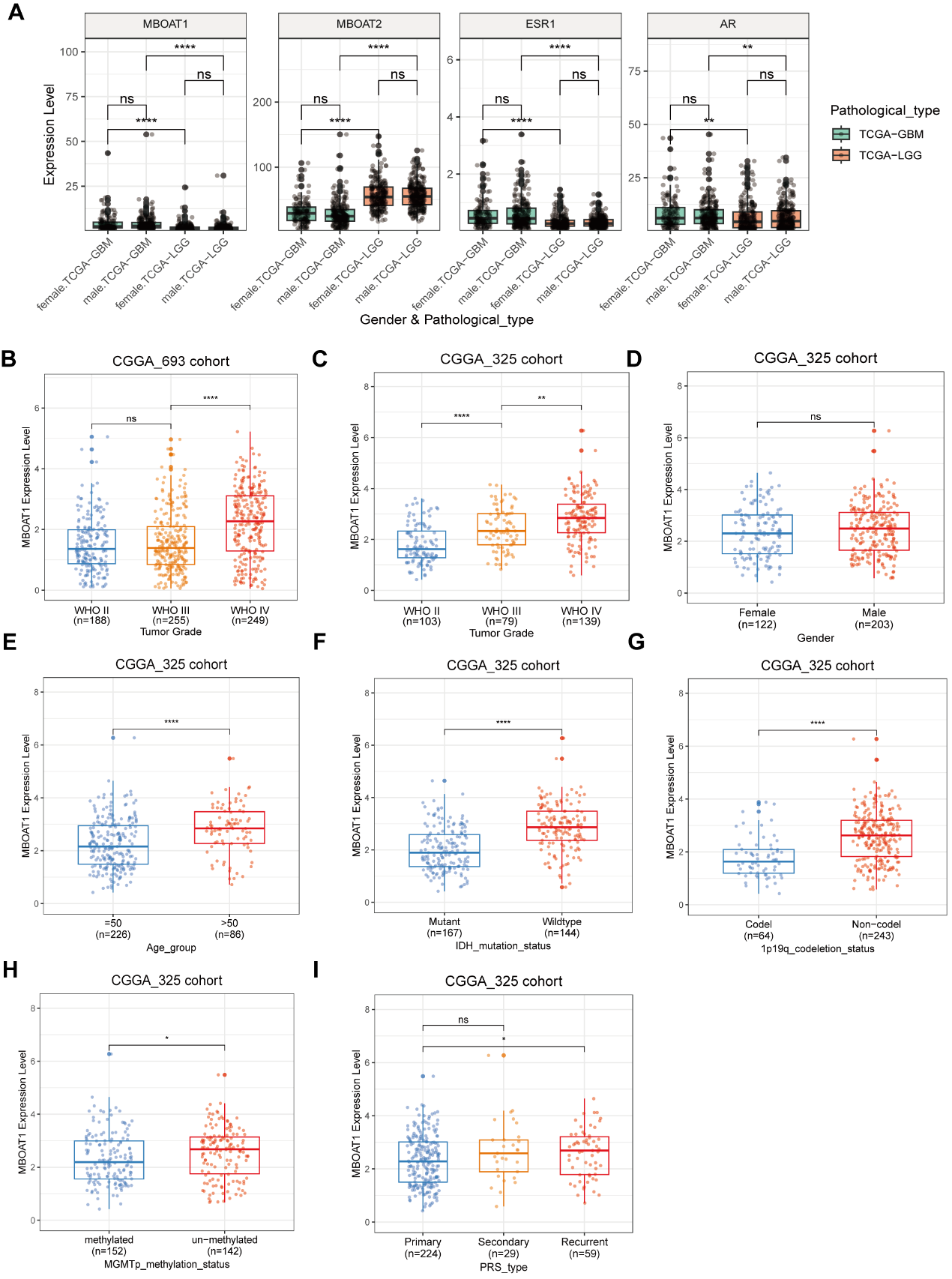


Figure.S2 Correlation of MBOAT1 with clinical variables. (**A**) Gender differences of MBOAT1/2, ESR1 and AR expression in LGG and GBM. (**B, C**) Association between MBOAT1 and WHO grade in CGGA cohorts. Box plots showing the association between MBOAT1 and various clinical variables in CGGA_325 cohort, including gender (**D**), age (**E**), IDH mutation status (**F**), 1p19q codeletion status (**G**), MGMTp methylation status (**H**), primary and recurrent subtype (**I**). Data are presented as mean ± SD. *P<0.05; **P<0.01; ***P<0.001; ****P<0.0001


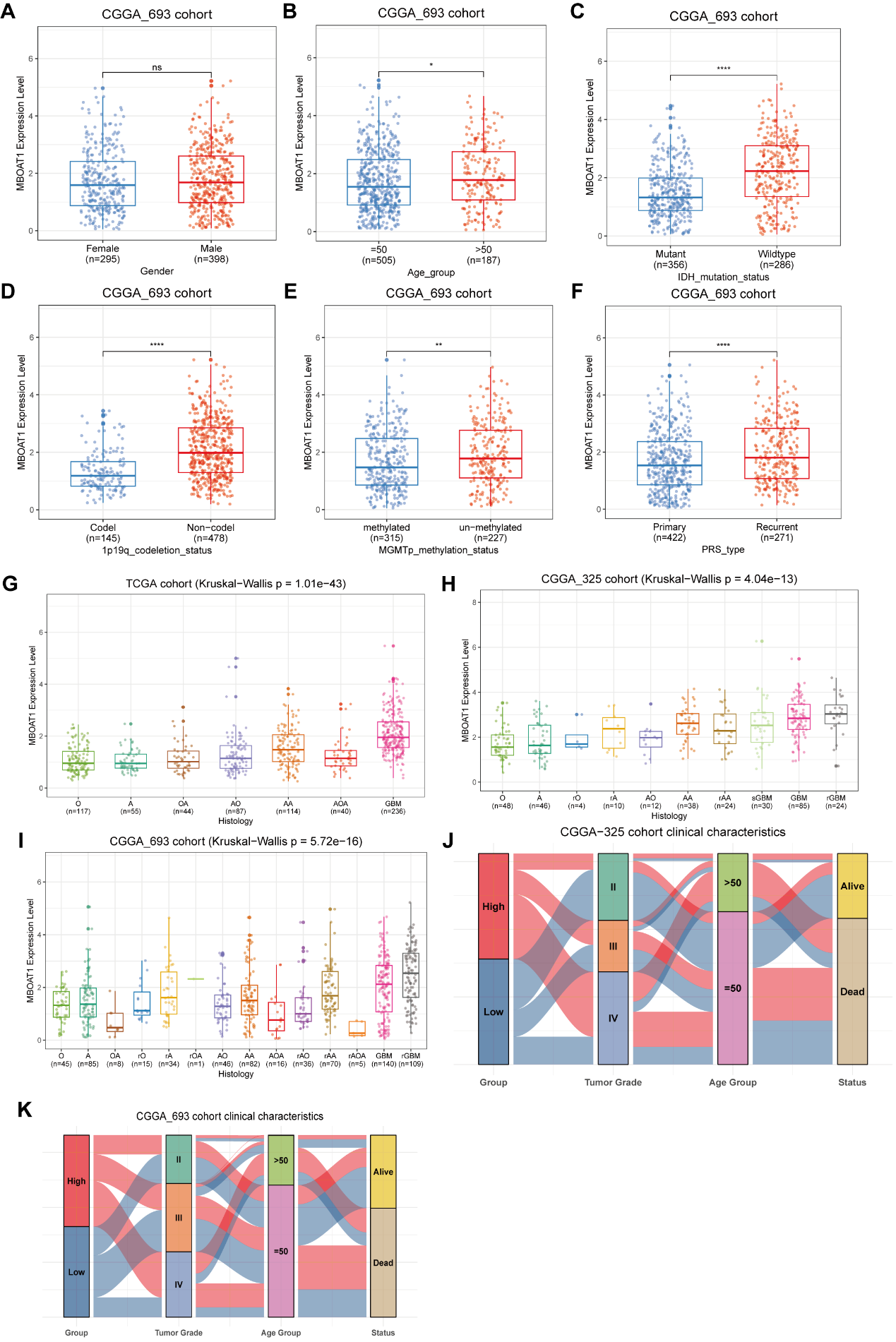


Figure.S3 Relationship between MBOAT1 and clinical variables. (**A-F**) Boxplots of association between MBOAT1 and various clinical variables in CGGA_693 cohort. (**G-I**) The association between MBOAT1 and histology in TCGA and CGGA cohorts. (**J**, **K**) The Sankey diagram demonstrates the relationships between clinical outcomes and several clinical features in CGGA cohorts. Data are presented as mean ± SD. *P<0.05; **P<0.01; ***P<0.001; ****P<0.0001


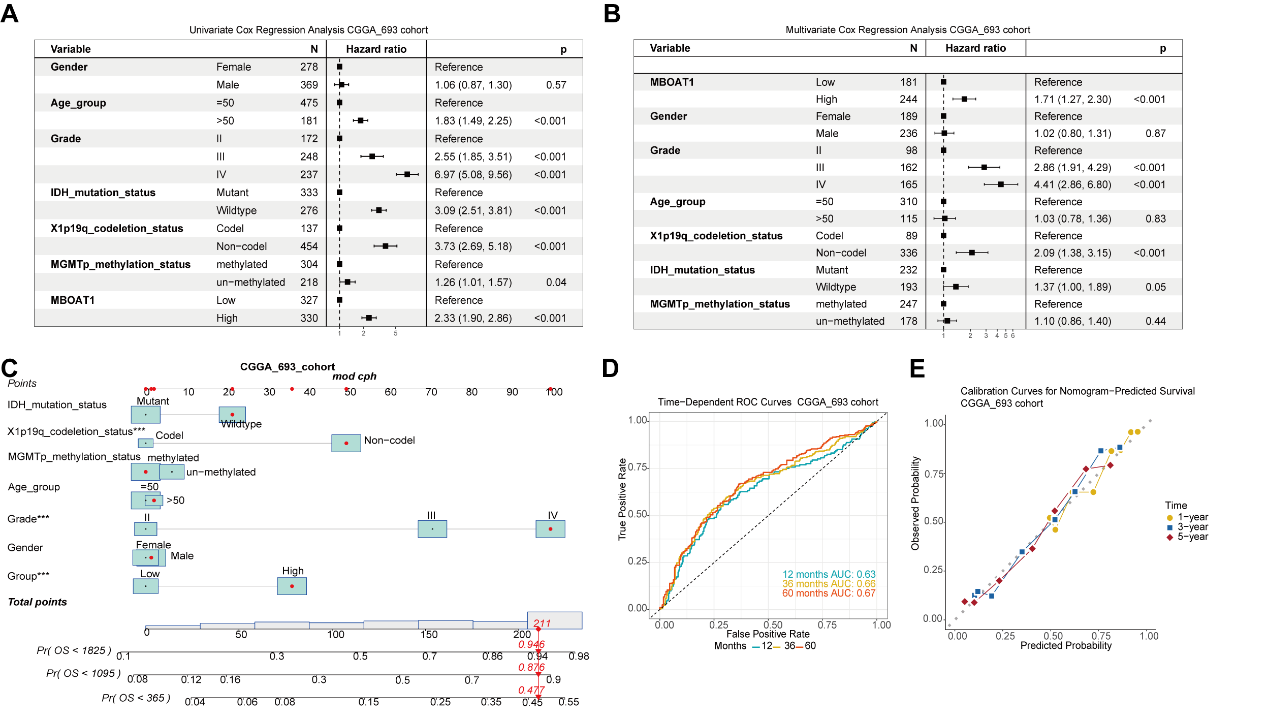


Figure.S4 The prognostic value of MBOAT1 expression in CGGA_693 cohort. (**A**) Univariate Cox regression analysis of clinical pathological characteristics in CGGA_693 cohort. (**B**) Multivariate Cox regression analysis of clinical pathological characteristics in CGGA_693 cohort. (**C**) A nomogram was utilized to predict the OS of glioma patients from the CGGA_693 cohort. (**D**) ROC curves to evaluate the predictive ability of MBOAT1 on survival time of patients in CGGA_693 cohort. (**E**) Calibration curves of the nomogram for predicting 1, 3, and 5years in in glioma patients from CGGA_693 cohort.


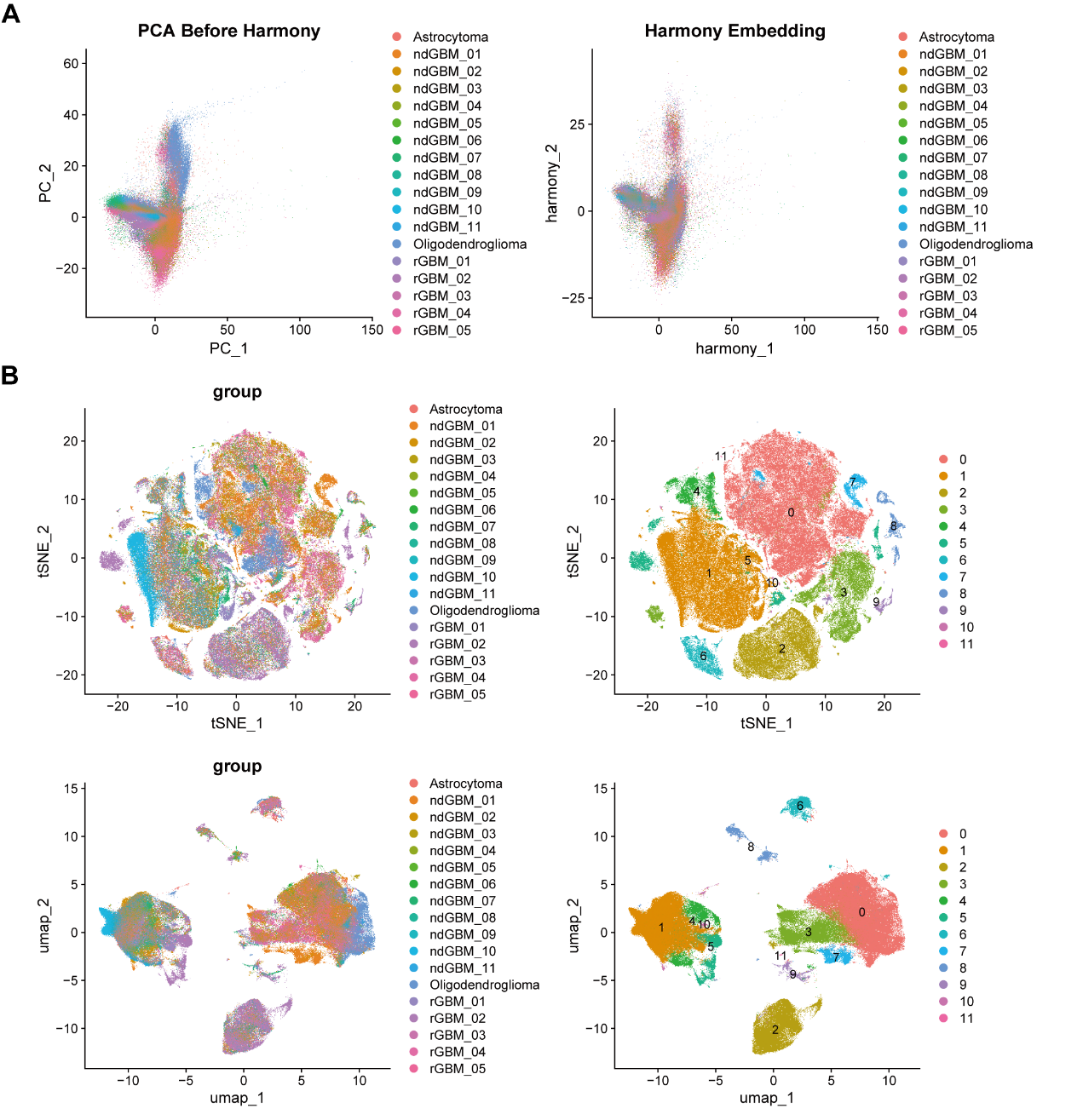
Figure.S5 Batch effect correct and clustering. (**A**) PCA plot of the batch effect before and after harmony. (**B**) The UMAP and tSNE map shows cell clusters in different patients. *P<0.05; **P<0.01; ***P<0.001; ****P<0.0001


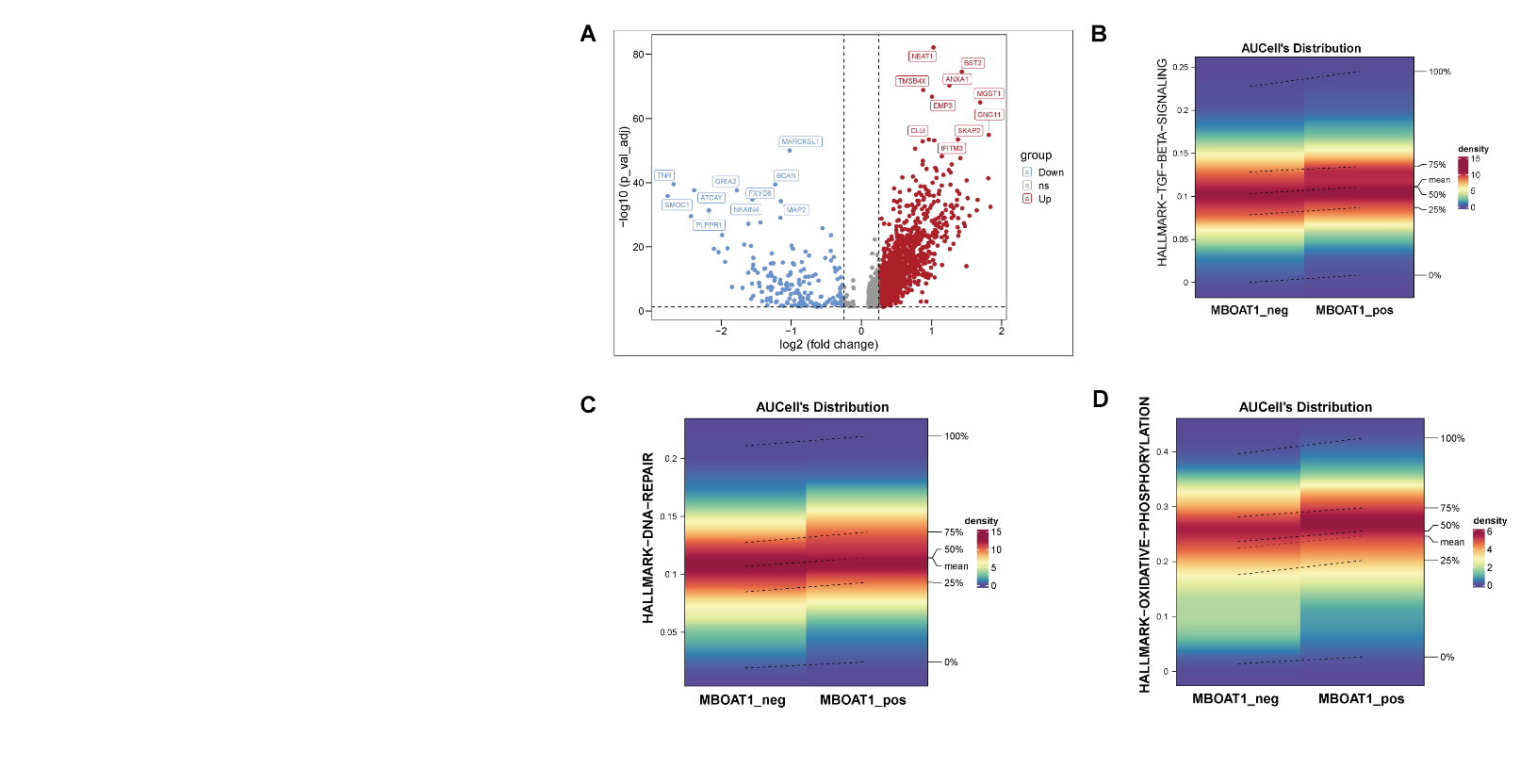


Figure.S6 Diferential analysis and AUCell scoring. (**A**) Volcano plot displays the differentially expressed genes in MBOPAT1 positive and negative groups. (**B**) Density plot shows differences in the TGF-β pathway between two groups. ****P<0.0001


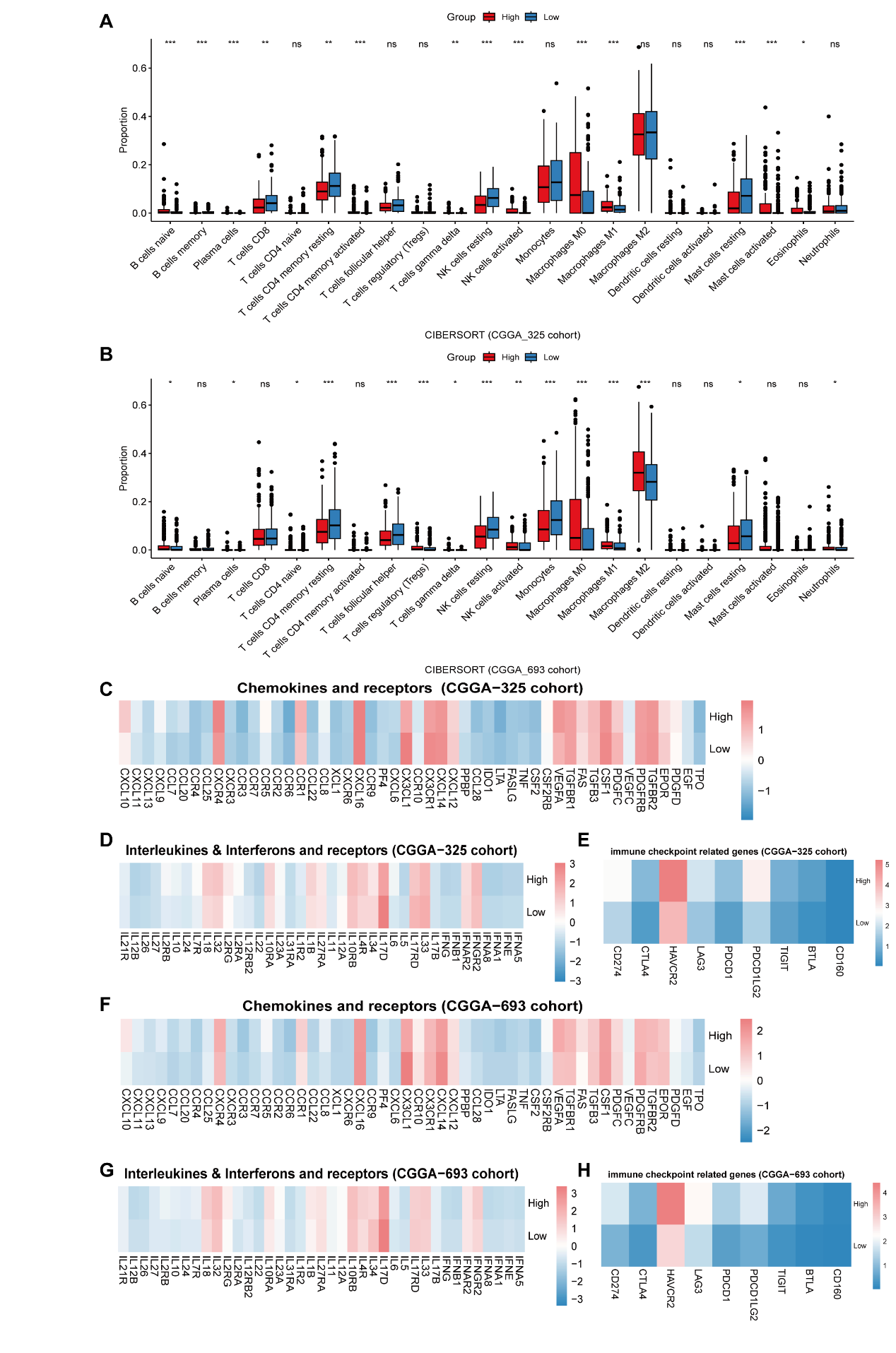


Figure.S7 MBOAT1 regulates tumor infiltration of immune cells in CGGA cohorts. (**A, B**) The box plot shows the frequency of TME infltrating cells between high and low MBOAT1 groups in CGGA_325 and CGGA_693 cohorts. (**C-H**) The heatmap shows variations in mRNA expression of chemokines, interleukins, other cytokines and immune check points related genes between high and low MBOAT1 groups in CGGA cohorts. *P<0.05; **P<0.01; ***P<0.001; ****P<0.0001


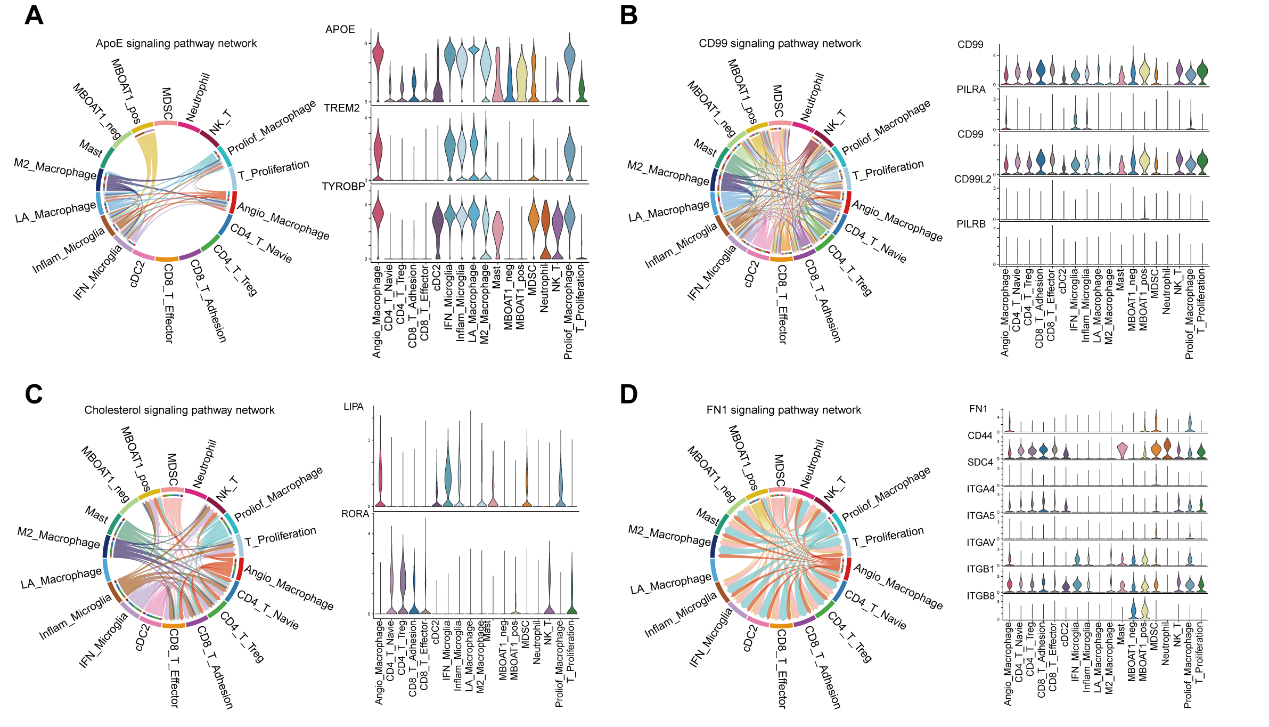


Figure.S8 Cellchat pathway analysis between positive and negative MBOAT1 glioma cells. (**A-D**) Chord diagrams and violin plots demonstrate differential distribution of receptor-ligand pairs.


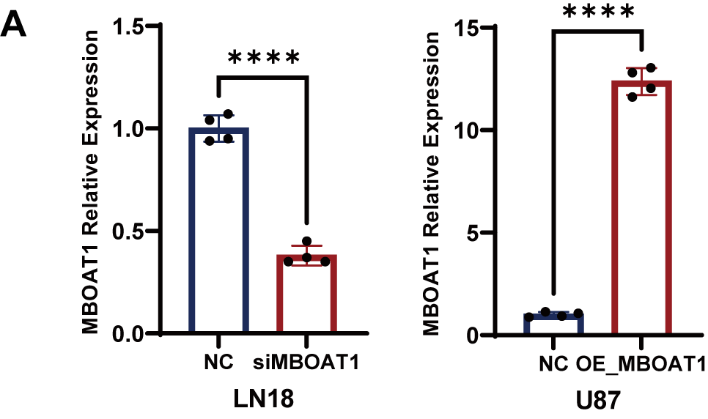


Figure.S9 Transfection efficiency assay. (**A**) Bar plots depict plasmid overexpression efficiency and siRNA knockdown efficiency in LN18 and U87 cells. Data are presented as mean ± SD. *P<0.05; **P<0.01; ***P<0.001; ****P<0.0001
